# Modeling human activity comprehension at human scale: Prediction, segmentation, and categorization

**DOI:** 10.1093/pnasnexus/pgae459

**Published:** 2024-10-11

**Authors:** Tan T Nguyen, Matthew A Bezdek, Samuel J Gershman, Aaron F Bobick, Todd S Braver, Jeffrey M Zacks

**Affiliations:** Department of Psychological and Brain Sciences, Washington University in St. Louis, St. Louis, MO 63130, USA; Department of Psychological and Brain Sciences, Washington University in St. Louis, St. Louis, MO 63130, USA; Department of Psychology and Center for Brain Science, Harvard University, Cambridge, MA 02138, USA; Computer Science and Engineering, Washington University in St. Louis, St. Louis, MO 63130, USA; Department of Psychological and Brain Sciences, Washington University in St. Louis, St. Louis, MO 63130, USA; Department of Psychological and Brain Sciences, Washington University in St. Louis, St. Louis, MO 63130, USA

**Keywords:** event cognition, segmentation, action perception, computational modeling

## Abstract

Humans form sequences of *event models*—representations of the current situation—to predict how activity will unfold. Multiple mechanisms have been proposed for how the cognitive system determines when to segment the stream of behavior and switch from one active event model to another. Here, we constructed a computational model that learns knowledge about event classes (event schemas), by combining recurrent neural networks for short-term dynamics with Bayesian inference over event classes for event-to-event transitions. This architecture represents event schemas and uses them to construct a series of event models. This architecture was trained on one pass through 18 h of naturalistic human activities. Another 3.5 h of activities were used to test each variant for agreement with human segmentation and categorization. The architecture was able to learn to predict human activity, and it developed segmentation and categorization approaching human-like performance. We then compared two variants of this architecture designed to better emulate human event segmentation: one transitioned when the active event model produced high uncertainty in its prediction and the other transitioned when the active event model produced a large prediction error. The two variants learned to segment and categorize events, and the prediction uncertainty variant provided a somewhat closer match to human segmentation and categorization—despite being given no feedback about segmentation or categorization. These results suggest that event model transitioning based on prediction uncertainty or prediction error can reproduce two important features of human event comprehension.

**Significance Statement**

We directly compared two accounts of the control processes governing how event representations in the brain are updated. A dominant account holds that event representations are updated when prediction error spikes. Another account proposes that event representations are updated when prediction uncertainty spikes. The two accounts reflect two modes of control, but they have been difficult to distinguish because error and uncertainty are highly correlated. Here, our naturalistic corpus allows them to be dissociated. This major scientific advance was enabled by a technical accomplishment: A large-scale activity corpus combined with a computational architecture that can handle full-scale activity, represented in a format approximating outputs of mid-level human visual processing, such that models’ outputs could be directly compared with human behavior.

## Introduction

To act effectively in a dynamic, complex environment, humans and other species use memory and knowledge to predict how activity will unfold over time (1–3). Knowledge about event classes can be encoded in structured representations called *event schemas* (4–6). One effective means to predict the near future is to use event schemas and sensory information to construct and maintain an internal model of the current situation (an *event model*). For example, if one is serving a cup of coffee, an event model can help to predict that the person receiving the coffee will pick it up, and how they will move to do so. However, for such a model to be effective it needs to *transition* when one thing ends and the other begins—once the coffee has been taken up, a coffee-serving model is unhelpful (7). How does a cognitive system effectively and efficiently decide when to transition its event models? One possibility is that the system learns to identify clusters of highly connected states of the world and updates at transitions from one cluster to another (8). An additional possibility is that the system simply tracks when the current state changes dramatically and updates when this happens (9). Latent cause inference models (10, 11) propose that the system learns latent classes that generate observable states and evaluates the probability of all latent classes at any moment based on observable states; event boundaries correspond to abrupt shifts in the probability distribution over latent classes. Inspired by latent cause models (10, 11), we propose that human comprehension starts from an assumption that experiences are generated by a series of unobservable latent classes. At each moment, the comprehension system holds one hypothesis about which class is most likely to have generated the current observed information. This inferred latent class is combined with instance-specific information to form the current event model. One possibility is that the current latent class is evaluated continuously (10, 12); however, latent class inference is computationally expensive so it is likely that latent class inference is gated. One possible gating mechanism is to monitor the system's predictions and transition when prediction quality degrades (11, 13). In particular, two aspects of prediction quality would seem to be good candidates for controlling event model transitions: spikes in prediction error (7, 14) or in prediction uncertainty (13, 15). Prediction error occurs when there is a misalignment between what the current event model predicts and what happens. In contrast, prediction uncertainty, what was called unpredictability in (13), occurs when the current event model is not able to make confident predictions. Differentiating between error-based and uncertainty-based mechanisms of event model updating can reveal how a cognitive system flexibly relies on different types of information to detect event boundaries and allocate attention (13). We set out to evaluate these two gating mechanisms in this paper and to compare them to a more computationally expensive continuous inference algorithm. There are also other candidate gating mechanisms for event model updating (8, 10, 11); here, we focus on these two because (ⅰ) prediction error is the dominant mechanism in current theories and (ⅱ) prediction error and prediction uncertainty can be straightforwardly compared with the ungated model, and to each other, in the structured event memory (SEM) architecture.

Models of comprehension in language (16) and visual perception (17, 18) have simulated predictive processing by training it to predict the next state on each timestep. One study (19) trained a gated recurrent neural network (RNN) using this one-step prediction task on a simplified sequence of human motion tracking data and found that the model's prediction error was associated with event boundaries. To the best of our knowledge, there has not been an attempt to relate a model's prediction uncertainty with event boundaries. The SEM (12) model extends neural network models of event cognition by representing different *event schemas* by separate RNN weight matrices, and each activation of an event schema constitutes an *event model*. This approach is contrasted with another line of research that represents all event schemas in a single network (20–23). Sharing the same network means that event dynamics across event types are learned jointly; thus, the network can benefit from commonalities across event types. Having separate RNNs deprives SEM of the ability to learn commonalities across event types. We addressed this in SEM-2.0 by initializing new schemas from weights of a single RNN trained on all seen activities (see below). SEM is tasked to predict the next state on each timestep based on its current event model. This raises the question of how to select the most appropriate event schema at each timestep. Bayesian inference has been widely proposed as a mechanism to infer event type given observed sensory sequences (10, 11, 23). In SEM, approximate Bayesian inference is used to determine which event schema is currently active based on observed scene transitions; this process is called event schema inference. The initially published SEM architecture evaluated all active and inactive schemas on every timestep. Here, we developed two variants of the SEM architecture that gate the schema inference mechanism based on either prediction error or prediction uncertainty. Thus, we were able to test candidate mechanisms for controlling event model transitions with architectures that are more computationally tractable and more biologically plausible.

In SEM, event schemas are learned by adjusting weight matrices based on observed scene transitions. As an event schema is learned from sequences of scenes, it comes to represent schematic knowledge associated with a particular class of event. Given finite learning experiences, there is uncertainty about the schematic knowledge. This kind of uncertainty is referred to as model uncertainty or epistemic uncertainty in Bayesian deep learning (24); it leads to uncertainty in predictions. (Another type of uncertainty concerns which event schema is appropriate to the current moment—in other words, which latent cause is responsible for the current observation (10). Although latent cause uncertainty influences prediction uncertainty (11) in full Bayesian inference, SEM approximates the distribution with a single high probability latent cause; thus, latent cause uncertainty does not influence prediction uncertainty in SEM.) Model uncertainty can lead to uncertainty about at least two salient features of activity: temporal dynamics (what comes next) and component structure (what are the components that will occur as part of a particular unit). For example, imagine Sarah is at a friend's house watching him making a special ice latte. Having seen baristas make iced lattes before, she is familiar with many aspects of the general process—boiling water, making an espresso shot, preparing ice, and steaming milk. However, having never seen this type of latte before, she is relatively uncertain about the specific sequence—whether the espresso should be added to the cup of ice before or after the steamed milk. In addition, this kitchen includes features that are new to her: a sophisticated espresso machine and a unique milk frother. For these devices, she might have greater uncertainty about their components—how to operate the espresso machine, which settings to use, and whether the milk frother needs any special preparation. Here, we evaluated transient increases in this type of uncertainty as a potential mechanism for gating the updating of event models and contrasting this with gating based on prediction error.

In naturalistic activity, prediction error and prediction uncertainty are correlated—when you are highly uncertain about what follows the keynote talk, your prediction is also more likely to be wrong. Also, naturalistic activity is characterized by rich correlations among perceptual features such as body movement and conceptual features such as interactions with objects. To generalize to real-world comprehension, it is important to include naturalistic structure in the modeling environment. To address these central features of naturalistic comprehension, we first adapted the SEM architecture so that it could be trained on the multi-angle extended three-dimensional activities (META) corpus, which is a 25-h corpus of richly annotated recordings of everyday activity (25). These recordings allowed us to directly compare the model's output to human performance on a moment-by-moment basis. We refer to the adapted model as SEM-2.0. We then developed variants of SEM-2.0 that gate the schema inference mechanism based on uncertainty (*uncertainty-SEM*) or prediction error (*pe-SEM*). We ran simulations to address two major questions: First, does a model that uses event representations to predict the unfolding of human activity (SEM-2.0) spontaneously identify human-like event boundaries and event categories? Second, does adding more biologically plausible gating mechanisms, reflecting prediction uncertainty (uncertainty-SEM) or prediction error (pe-SEM) cause the model to more closely approximate human behavior? The main difference between the two model variants and SEM-2.0 is that SEM-2.0 triggers the inference over all possible event schemas at every timestep, whereas uncertainty-SEM and pe-SEM only trigger the inference when a threshold for prediction error or uncertainty is reached.

**Fig. 1.**
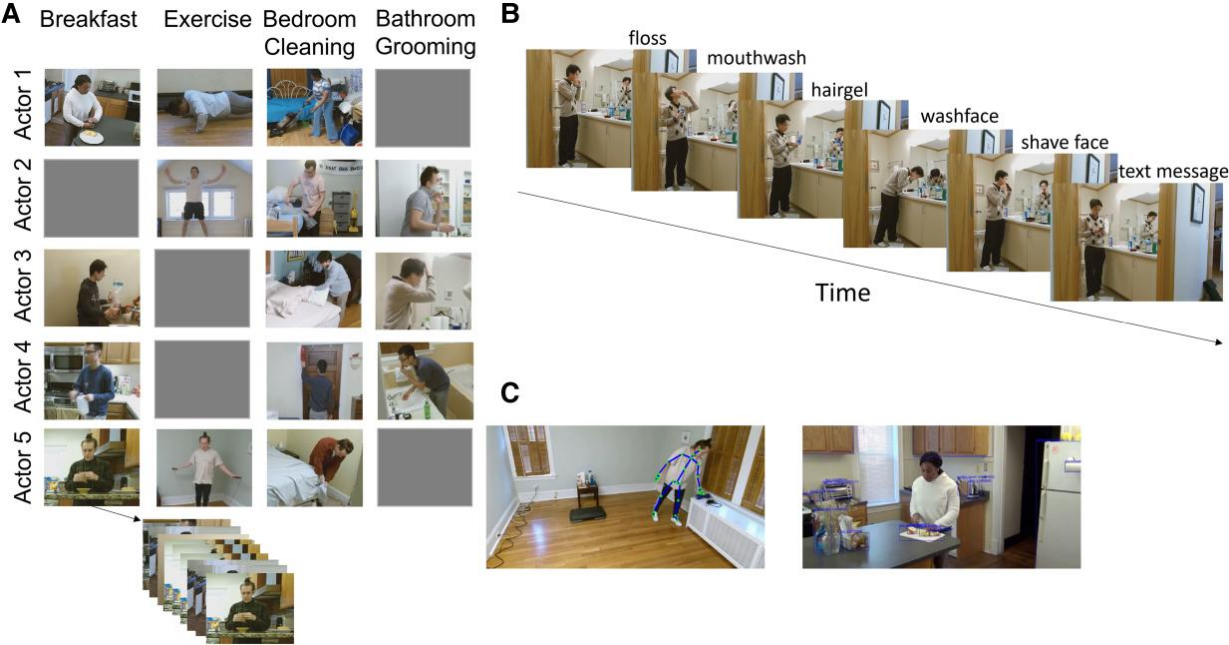
Structure of META dataset. A) Five actors performed four types of activities; each actor performed three out of four activity types. Each cell represents 10 unique recordings for each activity type. For example, actor 5 performed 10 unique sequences of actions pertinent to making breakfast. Gray cells indicate that an actor did not perform a particular activity type. For example, actor 1 did not perform the personal grooming activity. B) An example sequence of actions pertinent to personal grooming. C) Skeleton joint positions (left) and object location and identity (right) were extracted for all frames of a sequence. (The illustration only shows either skeleton or object for visualization purposes; in reality, both types of features were extracted for each frame.)

An important feature of the current work is that the models were trained on a large, rich corpus of recordings of live human activity (Fig. 1), such that the performance of the models could be compared directly to human perceptual and categorization judgments. In what follows, we first describe the model architectures and the dataset used to train and evaluate the models. Then, we present the results comparing the models’ segmentation and categorization agreement with human judgments on 3 h 39 min of validation activities, in order to compare how the model variants learned, segmented activity, and categorized incoming scenes. Finally, we assess how each model variant agrees with human segmentation and categorization to evaluate the candidate mechanisms of event model transitioning.

## Results

The three models of event comprehension were trained and tested on the META stimulus set, a corpus of naturalistic activities (25). In each activity, an actor performed a series of 6 to 7 scripted actions in a realistic environment (Fig. 1). Because the visual and semantic features processed in mid-level human vision may be the building blocks from which event representations are constructed (26), we used a combination of human coding and computer vision methods to generate a rich set of these features for each scene and used the generated vectors as input to the models. Specifically, from three-dimensional joint position recordings, we calculated features of body pose, velocity and acceleration, as well as inter-hand distance, velocity, and acceleration. To represent the semantic meanings of interactive objects in the activities, we annotated bounding boxes that tracked the positions of objects, then used a language model (GloVe (27)) trained on a large text corpus (28) and translated the name of each object to a vector embedding. We then computed a weighted vector representation of the objects closest to the actor's right hand and the mean vector representation of all objects currently present in the scene. Principal component analysis reduced a set of 253 input features (object appearances, object disappearances, mean frame-to-frame change in pixel luminance values, skeletal motion features, object semantic features) to a set of 30 features that we presented as *input scene vectors* to three models. The models were trained to take in the running sequence of these reduced representations and to predict the next timepoint in the sequence, one-third of a second later.

### Modeling human activity at scale

**Fig. 2.**
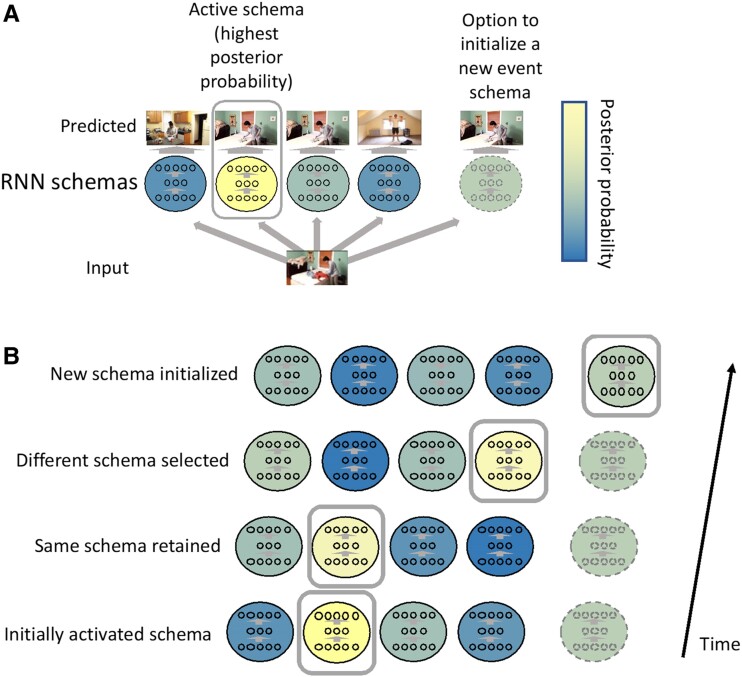
Overview of SEM architecture. A) In this hypothetical example, SEM is in the process of training and has generated four event schemas (four sets of RNNs’ weights). At each time step, the RNNs predict the current input scene vector; then, prediction errors are computed and converted to likelihoods, which contribute to posterior probabilities. Based on the posterior probabilities, SEM keeps the active event schema active, activates another schema in its library, or spawns a new event schema (depicted as an RNN with a dotted outline). The resulting active event schema updates its weights by backpropagating its prediction error. B) A potential sequence of outcomes. On the first two timesteps, the currently active schema is retained. On the third timestep, SEM switches to a different previously learned schema. On the fourth timestep, SEM initializes a new schema. (Not shown: SEM separately evaluates the probability of the current schema based on the current RNN's hidden unit values and based on re-initializing the RNN's hidden unit values. If resetting the hidden unit values is found to be more valuable, they are reset. This allows SEM to model, for example, washing a plate and then immediately washing a second plate.)

We will first describe the original SEM model (SEM-1.0) and then our modified SEM (SEM-2.0) and the two variants derived from SEM-2.0: uncertainty-SEM and pe-SEM. The core architecture of the SEM-1.0 model is depicted in Fig. 2. This model represents each event schema as an RNN's weight matrices and each event model as an RNN's weight matrices and hidden unit activations. SEM-1.0 uses an approximate Bayesian inference (clustering) process to assign incoming scene vectors to event schemas; thus, each scene vector has an *event label* (event schema's name). On each time step, a currently active RNN is presented with previous input scene vectors and predicts the current scene vector. The clustering process then compares the posterior probability of the active RNN's prediction, relative to posteriors from all other RNNs and then either (ⅰ) retains the current RNN, (ⅱ) activates a different RNN from the library, (ⅲ) retains the same RNN, but resets its activation values, or (ⅳ) spawns and activates a new RNN. We will refer to 2–4 as transitioning event models, and an *event boundary* is operationalized as a transition from one RNN to another RNN. SEM-1.0 utilizes several hyperparameters: *stickiness*, the tendency to keep the active model (to ensure temporal coherence in events); *concentration*, the tendency to spawn new models; and *learning rate* to update RNNs’ weight matrices.

SEM-1.0 (12) was able to account for structure in several small datasets, and it deployed new event schemas effectively, such that event schemas were frequently reused. However, when we applied SEM-1.0 to the META corpus dataset, we observed that the model activated only a small number of its event schemas across all 22 h of activities. Three modeling assumptions led to this ineffective use of resources. First, newly spawned-event schemas were initialized to random weights; this disadvantages new event schemas as they lack broad “general knowledge” about feature co-occurrence and dynamics. In SEM-2.0, we initialized newly spawned event schemas with weights from a single RNN that was trained on all scene vectors up to that point in time. The single RNN's weights serve to capture dynamics common across events. Second, the process SEM-1.0 used to assign prior probabilities to schemas was a sticky Chinese Restaurant Process (sCRP) (29). This process is a commonly used prior distribution that has a “rich-get-richer” property (30)—a small number of large classes accounts for most observations. In SEM-1.0, this property caused most timepoints to be assigned to only a small number of event schemas. Although “rich-get-richer” might be appropriate to some clustering applications, this property might not be desirable in applications where a more balanced prior distribution is appropriate (30, 31). In SEM-2.0, we instead used a uniform prior distribution, while retaining the stickiness and concentration parameters. Third, in SEM-1.0 active schemas made predictions about the current scene from the previous scene vectors, but inactive schemas were asked to make predictions from a random vector. This approach reduces model training and inference time, but it puts inactive schemas at a disadvantage. SEM-2.0 provides all schemas with input scene vectors from previous timepoints (see Materials and methods for details) to make predictions.

### Gating updating based on prediction error or prediction uncertainty

SEM-2.0, like SEM-1.0, infers event schemas for every incoming scene (timestep). In this schema inference process, SEM-2.0 asks all event schemas to predict the current scene given previous scenes (Fig. 2A), which is computationally expensive and biologically implausible. A more efficient and realistic approach is to make predictions based only on the current active schema and to use prediction quality as a gating signal to decide when to evaluate alternative schemas. We evaluated two candidate gating mechanisms: prediction error and prediction uncertainty; the models implementing each of these mechanisms are referred to as *pe-SEM* and *uncertainty-SEM*. At each timestep, each of the two variants used their active event schema to make a prediction; then, prediction error and prediction uncertainty were computed. Prediction error was operationalized as the Euclidean distance between an observed scene vector and the RNN's prediction. Prediction uncertainty was operationalized as variability in RNN predictions across perturbations of the RNN weights (epistemic uncertainty (24)), which we implemented by applying random dropout to the RNN repeatedly and generating predictions. Specifically, each RNN generates 32 different predicted scene vectors, with each prediction generated by different weight matrices derived by randomly dropping out weights of the RNN. The variance of these predicted scene vectors approximates prediction uncertainty induced by uncertainty about RNN's weights (32). In pe-SEM, if prediction error (pe-SEM) exceeds the prediction error threshold, the event schema inference process is triggered to determine the most probable event schema; otherwise, the current event schema is assigned to the current scene. Similarly, in uncertainty-SEM, if prediction uncertainty exceeds the prediction uncertainty threshold, the inference process is triggered. Thus, the difference between SEM-2.0 and the other two variants is that SEM-2.0 triggers the schema inference process at all timesteps, while the other two variants trigger the schema inference process when prediction error or prediction uncertainty is high. The schema inference process is the same for all three models.

From 128 activities (total duration: 21 h 43 m, range: 5 m 35 s to 19 m 16 s, mean: 10 m 11 s), activities were split into a training set of 108 randomly selected activities (18 h 4 m) and a validation set of the remaining 20 activities (3 h 39 m). In contrast to the common practice with deep learning models of interleaving learning with repeated presentation of stimuli (33), SEM-2.0, uncertainty-SEM, and pe-SEM encountered and learned each training activity only once, watching the whole activity before moving to the next activity. Even though activities do not repeat, there are multiple instances of the same event type spanning across different activity. For example, actor A performs “making a bagel” in activity 1 and actor B performs “making a bagel” in activity 2. This training regime approximates the structure of human naturalistic learning, in which each event is experienced only once, but similar events and event sequences recur. After training on each activity, the validation set was tested with learning turned off.

### All three models learn to predict naturalistic scene dynamics

**Fig. 3.**
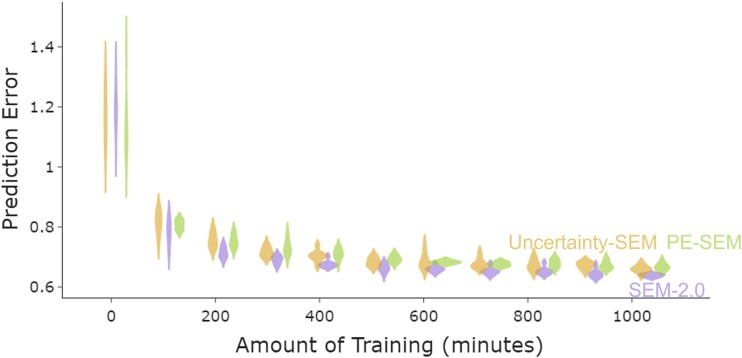
All models learn to predict naturalistic activity, with SEM-2.0 having the lowest prediction error. Each purple, goldenrod, or green violin plot is a distribution of prediction errors for eight different simulations of SEM*-*2.0, uncertainty-SEM, and pe-SEM, respectively. Validation prediction error of SEM-2.0, uncertainty-SEM, and pe-SEM decreases over time. At the end of the training, SEM-2.0 has the lowest prediction error, whereas uncertainty-SEM and pe-SEM have similar prediction errors.

Mean prediction errors for validation activities of all model variants decreased throughout training (Fig. 3). At the end of training (>900 min), SEM-2.0, which performs the inference over event schemas to select the most predictive event schema at all timepoints, had lower mean prediction error for validation activities compared with pe-SEM (two-sided t test, *t*-statistics = −7.96, *P*-value = 5.4e−08, and degree of freedom (df) = 30) and uncertainty-SEM (two-sided t test, *t*-statistics = −6.40, *P*-value = 2.2e−06, and df = 30). Uncertainty-SEM's and pe-SEM's prediction errors were not significantly different (two-sided t test, *t*-statistics = −0.85, *P*-value = 0.4, and df = 30).

To assess the impact of event knowledge partitioning, we created a *generic model*, composed of a single RNN that predicted the incoming scene vector from the last input scene vectors, and compared its mean prediction error to SEM-2.0's mean prediction error. This model used the same parameters as SEM-2.0 for its one event schema, but could not switch or spawn new event schemas. The generic model had a higher mean validation prediction error and was more susceptible to interference than SEM-2.0 (Fig. S15). We also created generic models with double and triple the number of RNN's hidden units. The double generic model had comparable prediction error with SEM-2.0, and the triple generic model had lower prediction error than SEM 2.0. During online comprehension, humans maintain event models which need to balance the tradeoffs between efficiency and accuracy. These results suggest that instead of learning a single large model that might be computationally expensive, learning multiple smaller models and transition between them could achieve similar level of accuracy while being more efficient.

### The models’ segmentation correlates with human segmentation, and updating based on uncertainty leads to more human-like segmentation than updating based on prediction error

A key test of the models is whether they generate human-like event boundaries for naturalistic stimuli. To compare SEM-2.0, uncertainty-SEM, pe-SEM to human performance on event updating, we used data from the META stimulus set (25). Normative event boundaries were collected from an online sample of participants. Participants were instructed to watch a randomly selected sequence and to press a button each time one meaningful unit of activity ended and another began. This task is widely used in the human event segmentation literature (34, 35). Each participant was assigned a grain of *coarse*, defined as the largest meaningful units of activity, or *fine*, defined as the smallest meaningful units of activity, and instructed appropriately. Participants could segment multiple videos. We collected 30 segmentations per grain per activity.

Our goal was to compare the performance of each model variant when it segmented in a range similar to that of our human participants. To that end, we conducted a hyperparameter search for each of the models to minimize the difference between the models’ number of boundaries and the median fine-grained event boundaries identified by humans (Fig. S10). We modeled human fine segmentation instead of coarse segmentation so that we would have more event boundaries per activity to model, increasing statistical power.

**Fig. 4.**
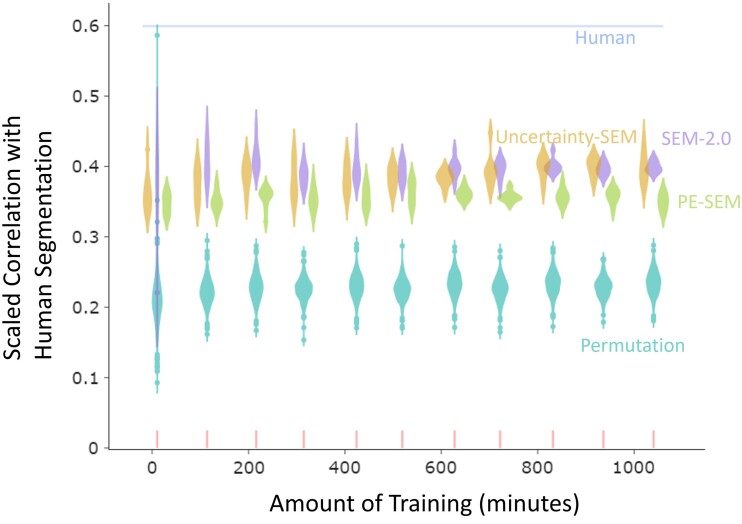
The three models’ segmentation agrees with that of human observers, even though they were never given feedback on segmentation. Updating based on uncertainty led to more human-like segmentation than updating based on prediction error. Scaled point-biserial correlations across all validation activities for SEM*-*2.0, uncertainty-SEM, pe-SEM, humans, and permutations across training. Each purple, goldenrod, or green violin plot is a distribution of point-biserial correlation for eight different simulations of SEM*-*2.0, uncertainty-SEM, and pe-SEM, respectively. Each light sea-green violin plot is a null distribution generated by shuffling models’ boundaries while preserving event lengths. The cornflower line is the median of the distribution of scaled point-biserial correlation between different human subjects and normative group segmentation. SEM-2.0's segmentation agreement with human segmenters was greater than expected by chance. Scaled point-biserial correlations for SEM-2.0 and uncertainty-SEM are not significantly different and are significantly higher than scaled correlation for pe-SEM.

To quantify human-to-human and model-to-human segmentation agreement, we calculated the proportion of human raters who segmented during each timestep and computed the point-biserial correlation between that normative human segmentation timeseries and (ⅰ) each individual human rater and (ⅱ) the models’ event boundaries. The possible range of this correlation depends on the number of event boundaries; thus, comparing correlations for two segmenters who identify different numbers of event boundaries can be misleading. Therefore, we scaled the correlation (36) based on its minimum possible and maximum possible values, given the number of boundaries observed. We also assessed how likely the result would occur by chance by creating permuted boundaries: shuffling the models’ event boundaries while preserving event lengths (see Materials and methods). Then, we computed scaled point-biserial correlations between the models’ boundaries and human boundaries and between the permuted boundaries and human boundaries. As shown in Fig. 4, scaled point-biserial correlations for all models at the end of training (>900 min) were much larger than would be expected by chance (two-sided t tests, *t*-statistics are 61.59, 36.81, and 41.82, *P*-values are 2.80e−44, 6.48e−21, and 2.01e−41, and dfs are all 62 for SEM-2.0, uncertainty-SEM, and pe-SEM, respectively), and were almost halfway from the median of permutation-to-human to the median of human-to-human segmentation agreement (0.44, 0.46, and 0.32 for SEM-2.0, uncertainty-SEM, and pe-SEM, respectively). Scaled point-biserial correlations of SEM-2.0 and uncertainty-SEM were not significantly different (*t*-statistics = 0.23, *P*-value = 0.82, and df = 30), and both had higher scaled point-biserial correlations than pe-SEM did (two-sided t tests, *t*-statistics are 24.28 and 14.11, *P*-values are 6.3e−21 and 9.82e−12, and dfs are all 30 for SEM-2.0 and uncertainty-SEM, respectively) (Fig. 4). That is, the models segmented in a human-like fashion, with SEM-2.0 and uncertainty-SEM showing stronger agreement with human segmentation than pe-SEM.

The architectures of uncertainty-SEM and pe-SEM were identical except for the triggering mechanism. However, the concentration and stickiness hyperparameters that were needed to match human segmentation differed (Table S3): pe-SEM has smaller concentration and larger stickiness compared with uncertainty-SEM. When pe-SEM and uncertainty-SEM were run with the same concentration and stickiness settings, pe-SEM spawned more event schemas and had more event boundaries. This is because the likelihood of spawning a new schema or switching to an old schema is inversely proportional to prediction error. The differences regarding segmentation or categorization between the two model variants could be due to the difference in the triggering signals (i.e. prediction error or uncertainty) or to differences in the tendency to stick to the current event (stickiness) and the tendency to spawn a new event (concentration). To rule out the latter explanation, we compared uncertainty-SEM using hyperparameters matched to human segmentation with pe-SEM which had hyperparameters matched to those chosen for uncertainty-SEM. We also ran the converse comparison. These manipulations did not change the result: Uncertainty-SEM's boundaries still agreed with human normative boundaries more than pe-SEM's boundaries did (Figs. S16 and S18).

### The models’ categorization corresponds with human categorization, and updating based on uncertainty leads to more human-like categorization than updating based on prediction error

**Fig. 5.**
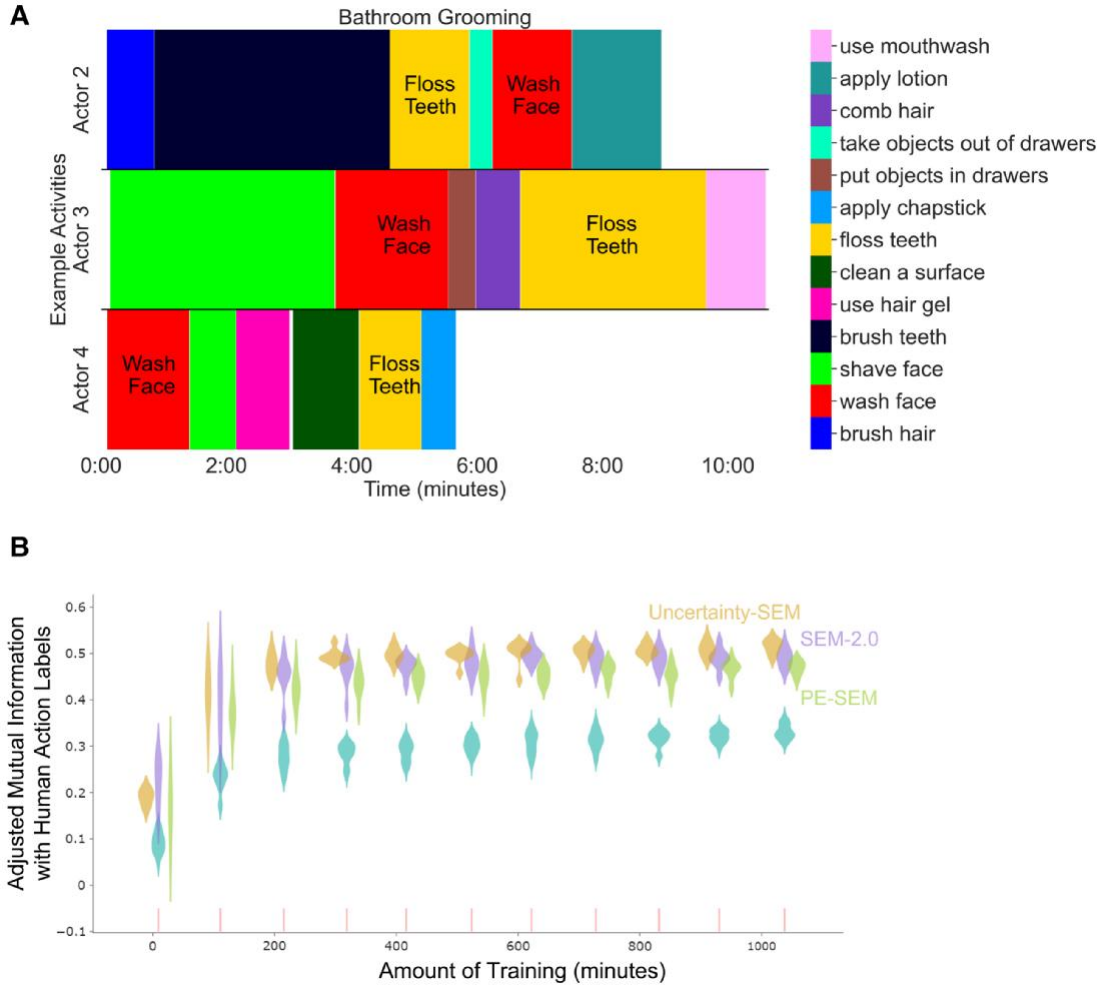
Three models’ categorization agrees with human categorization, despite never receiving feedback on categorization. A) Examples of human action labels for “bathroom grooming” activities. Each row is an example activity from a different actor performing these action sequences. Each color represents an action label. The *X*-axis indicates the time and duration of the action. Notably, actions varied in their order and durations across activities, as illustrated by the labeled “Wash Face” and “Floss Teeth” actions. B) Categorization agreement between three models’ event labels and human action labels. Each violin is a distribution of adjusted mutual information scores for eight different simulations of each model (purple for SEM-2.0, goldenrod for uncertainty-SEM, and green for pe-SEM). Light sea-green violins indicate distributions of adjusted mutual information scores between permutated event labels and human action labels. All models’ adjusted mutual information scores increase across training and remain significantly bigger than chance. Uncertainty-SEM's event labels agree with human action labels more than SEM-2.0's and pe-SEM's.

To comprehend an activity, one needs to not only capture its boundaries but also relate the current activity to previous knowledge. For each scene, the three models select an event schema to remain active or become active; this can be interpreted as an event label categorizing that scene. A model's event labels represent its categorization of a current moment as an instance of a previously learned activity. To evaluate the models’ ability to classify, we used the *script action labels* that were provided to the actors before recording each activity. We asked two human raters to watch videos of the activities and identify the beginning and end of each of the 6–7 scripted actions per activity. Agreement between raters was high, with a median discrepancy of 1.40 s between raters. Discrepancies were resolved by computing the mean of the time annotations. We compared human-rated action labels and each model's event labels. Figure 5A shows examples of these script action labels.

To quantify the models’ agreement with human action categories, we calculated the *adjusted mutual information* (37) between each model's event labels and the scripted action labels. Mutual information quantifies the information shared by the two partitioning (clustering) algorithms (model variants and humans partition input scene vectors into clusters) and thus can be employed as a categorization similarity measure. If models categorize input scene vectors in a human-like way, the mutual information between models’ event labels and script action labels will be high (see Materials and methods). The adjusted mutual information score corrects for the chance level of expected mutual information between two partitioning algorithms. We also assessed how likely the result would occur by chance by creating permuted event labels: shuffling the models’ events while preserving event lengths (see Materials and methods). This is the same shuffling procedure we performed to generate permuted boundaries (when events are shuffled, event boundaries are also changed). Then, we computed adjusted mutual information between models’ event labels and human action labels and between permuted event labels and human action labels. As shown in Fig. 5B, the adjusted mutual information between each model's event labels and script action labels was all significantly higher than the permuted null distributions (two-sided t tests, *t*-statistics are 26.64, 31.67, and 23.55, *P*-values are 1.20e−35, 5.42e−40, and 1.26e−32, and dfs are all 62 for SEM-2.0, uncertainty-SEM, and pe-SEM, respectively). Qualitatively, an examination of the correspondence between each model's schemas and script action labels revealed that event schemas generalized across actors and environments: The same schema was often activated for the same script action performed by different actors in different environments (Fig. S5).

To assess whether updating based on uncertainty or prediction error led to more human-like categorization, we compared SEM-2.0's categorization agreement with humans to those of pe-SEM and uncertainty-SEM. At the end of training (>900 min), adjusted mutual information with human action labels for uncertainty-SEM was significantly higher than pe-SEM (two-sided t test, *t*-statistics = 7.68, *P*-value = 1.45e−08, and df = 30); indeed, it had higher agreement than SEM-2.0 did (two-sided t test, *t*-statistics = 3.47, *P*-value = 1.61e−03, and df = 30). SEM-2.0 had significantly higher agreement than pe-SEM did (two-sided t test, *t*-statistics = 3.43, *P*-value = 1.78e−03, and df = 30). Uncertainty-SEM's categorization also agreed with humans more than pe-SEM's categorization in the two hyperparameter-matched regimes (Figs. S17 and S19), suggesting that the observed differences were not due to differences in hyperparameter values.

Together, the segmentation and categorization results suggest that an architecture controlling the inference process over event schemas through prediction uncertainty leads to more human-like behavior than a computationally intensive architecture that performs the inference process all the time or an architecture controlling the inference process by prediction error.

Note that perfect adjusted mutual information is possible only if two partitions have the same number of categories; if two partitions differ in the number of categories (i.e. if one is finer than the other), the best possible adjusted mutual information score is lower. Over training, the models develop more specific event schemas and finer-grained segmentation: By the end of the training, their mean event lengths were 29.47, 28.96, and 24.7 s for SEM-2.0, uncertainty-SEM, and pe-SEM respectively, suggesting that they were identifying sub-events of the scripted actions (mean length of 79.90 s). This lowers the adjusted mutual information score. To better understand how finer-grained segmentation affects the relationship between each model's evolving partitioning and human categories, we used two complementary categorization metrics, *purity* and *coverage* (38). This analysis confirmed that all three models’ categorization at the end of training captured sub-units of the action script labels (Fig. S7).

### SEM-2.0 and uncertainty-SEM produce flurries of updating at some event boundaries

In SEM-2.0's and uncertainty-SEM's performance, we noted “flurries” of event model transitions such that the model would often update multiple times in rapid success and then settle back into a stable state. These occurred more frequently for SEM-2.0 than for uncertainty-SEM and were not observed for pe-SEM. Figure S21A and B shows SEM-2.0's boundaries for two example validation activities. Figure S21C shows the distribution of elapsed durations between consecutive boundaries. The distribution is heavily right-skewed, and ∼36% of SEM-2.0's and 15% of uncertainty-SEM's pairs of consecutive boundaries have durations below 1 s, showing that both models make flurries of rapid transitioning within a short time. Even though we know that humans agree on where event boundaries are, we do not know whether it is the case that the brain experiences one boundary or a flurry of boundaries. Thus, SEM-2.0 and uncertainty-SEM make a novel prediction that the brain might sometimes experience a series of switches before settling into a new stable event model. This prediction extends existing theories by introducing the concept of transitional instability in event model formation.

### The generation and use of event schemas

One feature of the META dataset is that there are multiple instances of the same event class (e.g. there are multiple performances of “making coffee” spanning environments and actors). If the models successfully learn dynamics associated with event classes, we should expect them to reuse event schemas later in training. We observed that the models spawned new event schemas at a higher rate early in training and reused event schemas more often later in training (Fig. S20).

### Comparison with input-deprived models

To explore the contribution of motion features and semantic features on uncertainty-SEM's prediction, segmentation, and categorization, we created two input-deprived uncertainty-SEM versions: semantics-deprived and motion-deprived models (see Materials and methods) and compared them to uncertainty-SEM. Deprived models have higher prediction errors than uncertainty-SEM, with a large contribution coming from the respective deprived features. Full uncertainty-SEM had higher segmentation correlation with humans and categorization agreement with humans than did the two deprived models (Figs. S13 and S14).

## Discussion

Event models facilitate effective real-time responses to dynamic activity, planning for the future, and encoding for long-term memory (2, 26, 39–42). Previous modeling attempts have been conducted on stimuli that were highly abstract or simplified, and too brief to capture the variability and structure of naturalistic events (8, 12, 19). The META dataset (25) provides a more rigorous test for computational models of event comprehension by allowing for a model training and testing regime that resembles human learning experience, that humans never experience the same event twice but experience similar events of the same event class (e.g. similar “making bagel” events). The present simulations asked two critical questions about event comprehension. First, does encapsulating event dynamics in schemas, with schema selection governed by inferring latent structure in the dynamics of activity, facilitate learning the dynamics of naturalistic activity? Second, does gating updating based on prediction error or prediction uncertainty lead to more human-like segmentation and categorization? The results show that encapsulating schema knowledge improves the prediction of upcoming activity and reproduces two features of human behavior: segmentation and categorization. In addition, an uncertainty-based updating mechanism leads to more human-like event segmentation and action categories than gating based on prediction error does.

### Prediction

The model was trained to predict one-third of a second into the future based on a sequence of scene representations. The simulation input and output scene vectors were constructed to capture key properties of object and action processing in the primate visual system (43); we view these as a rough summary of the results of early- and mid-level visual processing. This strategy allowed for direct comparison of human and model event segmentation and categorization, enabled the model to generalize its schemas across actors and environments, and should facilitate generalization to other everyday activities as well as comparison to neurophysiological data.

The model's ability to learn is grounded in its hybrid architecture—a gated recurrent network to learn short-run event dynamics joined with the inference process over event schema to model transitions from event to event. These results are consistent with previous findings that RNNs are effective for sequential learning (16, 33, 44). Comparison models that did not incorporate event schema inference were able to achieve reasonable performance on the prediction task; however, they were less able to protect previous learning from interference (45, 46). SEM's segregation of event knowledge into multiple schemas distinguishes it from previous models of event cognition (8, 19–21), in which event dynamics was learned by a single neural network instead of a library of networks.

### Segmentation

In our two model variants, segmentation reflects the updating of the model's current event representation in response to prediction error or uncertainty. This instantiates the core principle of event segmentation theory (14). Crucially, the model was never provided with information about human segmentation; our finding that its segmentation corresponded with human segmentation occurred as a natural side effect of implementing the algorithm. The results are consistent with a small-scale demonstration using SEM-1.0 (12). All model variants produced better than chance level segmentation agreement with human participants when trained and evaluated on large-scale naturalistic activities. Further, uncertainty-SEM (and SEM-2.0) produced a significantly higher agreement with human segmentation than pe-SEM. Though the three models still did not correspond well to human segmentation, they could serve as a baseline for future modeling effort with naturalistic events.

### Categorization and generalization

All variants of SEM-2.0 generated event schemas that matched human action categories better than chance, again without being provided with information about human categories. Uncertainty-SEM produced more human-like categorization than pe-SEM or SEM-2.0. It generalized event schemas to performances by different actors in different environments, indicating that it could learn underlying event dynamics while smoothing surface features.

### Monitoring prediction quality: error and uncertainty

Prediction error (3, 7, 14, 42, 47) and prediction uncertainty (11, 13, 15) are two signals of the quality of a current event model's appropriateness. They are correlated but not at all identical. The current results have important implications for theories of event comprehension, including event segmentation theory, which posits that event models are updated at spikes in prediction error (14, 19, 42, 47, 48). They suggest that other metrics of prediction quality—specifically prediction uncertainty—should be considered as potential gating mechanisms for human event segmentation (11, 13, 49, 50). Benchmarking these two mechanisms against human performance required a large, naturalistic corpus of human activity recordings.

Given the proposed theoretical differences (13)—prediction error-based updating mechanism being reactive, responding to discrepancies in expectations, and prediction uncertainty-based updating mechanism being proactive, anticipating and preparing for future unpredictability—it is essential to delve deeper into the neural underpinnings associated with each mechanism in future research. Comparing these neural underpinnings will illuminate how the brain implements these distinct strategies. The current computational framework could help facilitate this task by providing estimates of moment-by-moment error, uncertainty, and boundary timecourses, enabling forward inference (51) with neuroimaging data.

### Limitations

The META corpus represents a substantial step forward as a testbed for modeling human event comprehension at scale (25). The activity recordings were processed to form representations intended to capture the core features of human body motion and object interactions that might be recovered by early- to mid-level human vision. This approach made the problem computationally tractable and made the results interpretable, but it is necessarily an approximation. The timestep was 1/3 s, which was chosen because this duration is not too long such that the motion and semantics features change drastically from one step to the next which makes it too hard to learn, and this duration is not too short such that the features change too slow which makes it too easy to learn. The specific decision is arbitrary and future work might explore other timesteps. In addition, the current SEM versions use RNNs to represent event schemas due to their ability to incorporate past information. However, the choice of a four-layer gated recurrent unit (GRU) is also arbitrary, and other architectures might yield different results. Prior work has shown limitations of similar RNNs in role-filler binding (52) and spatiotemporal tasks (53), suggesting the need for better representation of structural relations between entities. One approach is to augment an RNN with modules that are designed to progressively extract abstract representations such as in convolutional neural networks. Prior work (54, 55) used convolutional LSTMs (cLSTMs) as representation modules for the task of next-frame video prediction. A cLSTM is an enhanced version of the standard LSTM neural network, designed specifically for image sequence processing, such as in video analysis. Experiments have shown that cLSTMs consistently outperform traditional LSTMs in tasks that involve spatiotemporal data (53).

The current SEM versions do not have memory for schema updating, meaning that they must repeat inference when re-encountering similar scenes. A more computationally efficient alternative could involve storing previously inferred scene–schema pairs, allowing direct retrieval instead of repeated inference. This possibility has been the subject of recent research (56).

### Future directions

One important problem for future research is to account for the learning of hierarchical structure in activity (57). A sequence of small-scale events such as “browse the menu,” “call the server,” and “order meals” may occur reliably as part of a larger event such as “order food at a restaurant.” Behavioral (58, 59) and neuroimaging (60, 61) data provide evidence that human event comprehension represents such temporal hierarchy. The SEM architecture does not currently have a means to explicitly model the multiple grains and relations across grains. One limitation of the sticky uniform process (sUP) or the sCRP is that the prior for the next event is not influenced by the current event. Thus, one direction to explore is to extend the schema selection process to learn transition dynamics between events, effectively grouping sub-events belonging to a larger event together (62). Another strategy is to allow the model to learn events on multiple timescales simultaneously. One distinction between SEM and other modeling approaches (15, 20–22) is that these approaches represent different event types with the same RNN. This approach allows multiple timescales to be represented in the same latent space, where finer timescale dynamics are transitions between neighboring points in the latent space and coarser timescale dynamics are transitions between neighborhoods. It is worth evaluating the extent to which a single RNN could capture multiple timescales in future work.

Another central problem to be explored is the reuse of previously learned schemas to create a new one. For example, event “sell stock at a coffee shop” could include elements of event “sell stock” and elements of event “have coffee at a coffee shop.” One approach to such reuse is to represent events compositionally, decomposing components into elements and combining these components in a rule-like manner (63). Notably, Elman and McRae (20) demonstrated that a single, large neural network could combine elements learned in different events. The authors trained a neural network model on two different events: a person cuts food in a restaurant with a knife and a person cuts themselves with a knife and bleeds. The network was tested with a new event: a person was in the restaurant and cut themself, and it correctly inferred that the person bleeds, combining elements from the two learned events. The META corpus provides a more stringent testbed to evaluate the extent to which a single network could represent events compositionally.

## Materials and methods

### Model architectures and implementation

SEM-2.0 was adapted from SEM-1.0 to address its limitations when applied to our human-scale naturalistic dataset and to answer the question whether a model that uses event representations to predict the unfolding of human activity spontaneously identifies human-like event boundaries and event categories. Uncertainty-SEM and pe-SEM variants were adapted from SEM-2.0 to test whether adding a more biologically plausible gating mechanism to SEM-2.0 causes the model to approximate human behavior better, and whether prediction uncertainty or prediction error is a more suitable gating mechanism. We will describe SEM-1.0, SEM-2.0, and the two variants below.

The core architecture of the SEM-1.0 model has two main components: a library of RNN schemas and an approximate Bayesian inference module (12). The specific RNN architecture was a four-layer, fully connected neural network with GRUs, a leaky rectified linear activation function (leaky ReLU), and 50% dropout for regularization. An active RNN represents the active event model that humans maintain during online comprehension. We view the RNN with GRUs as a general-purpose engine for learning the short-run trajectories of events using stable hidden layers. Because we focused here on gating of the approximate Bayesian inference architecture, we adopted the RNN architecture directly from that of Franklin et al. (12) and did not vary it. The approximate Bayesian inference module clusters each incoming scene vector to an event schema by inferring which event schema generated the scene vector, using local maximum a posteriori (MAP) estimation (Fig. 1A). For each incoming vector, SEM-1.0 computes the likelihood that the vector belongs to each event schema by comparing event schemas’ predictions with the scene vector, with higher similarity indicating higher likelihood. SEM-1.0 assigns prior probabilities based on the sCRP. Priors and likelihoods are combined to compute posterior probabilities for all event schemas, and the incoming scene vector is assigned to the event schema with the highest posterior probability. Consequently, in this architecture, event boundaries are defined as selecting a new schema (or selecting to re-initialize the currently active schema). This inference mechanism over event schemas by estimating local MAP approximates Bayesian inference; exact Bayesian inference would require computations for all past clustering outcomes. However, comparisons between local MAP and more exact forms of Bayesian inference have shown their performance on certain problems to be highly similar (64).

In SEM-1.0 (12), there were three sources of bias (modeling assumptions) that created an imbalance in the relative activation of event schemas. One source of bias was that newly spawned event schemas were initialized to random weights. This initialization disadvantages new event schemas for the learning of naturalistic activities like the META corpus, because the environment is rich with general features and dynamics, such as where objects are typically found and how bodies can move, as well as event-specific information. To address this imbalance, we initialized newly spawned-event schemas with weights from an RNN that was trained on all scene vectors up to that point in time. Second, in SEM-1.0, the process used to assign priors to event schemas was the sticky sCRP, which assigns higher prior probabilities to event schemas that have more frequently been activated in the past. This led to the activation of a small number of event schemas for most time points and rarely activated newly spawned event schemas; therefore, in SEM-2.0, we replaced this process with the sUP, in which large and small clusters have equal probability; the sUP has been applied for event schema inference in another work (23). As in the sCRP, the sUP has a hyperparameter called stickiness that controls the tendency to remain in the currently active event and a hyperparameter called concentration that controls the likelihood of spawning new event schemas. Removing this “rich-get-richer” property helped SEM-2.0 to use event schemas more evenly (Fig. S2). Third, SEM-1.0 asked active schemas to make predictions about the current scene by feeding them scene vectors from previous timepoints while asking inactive schemas to make predictions by feeding them a random vector. This approach helped the authors circumvent a computational challenge because predictions from inactive schemas could be cached (because the random vector was constant, predictions were also constant) and used to compute likelihoods for the inactive event schemas. However, that approach placed inactive event schemas at a disadvantage because the input scene vectors to these event schemas were not informative to predict the current scene vector, while the input scene vectors to the active event schemas were the scene vectors from previous timesteps. Consequently, inactive event schemas were less likely to be selected and update their weights (since only the active event schema updates its weights at a specific timestep), resulting in only some initial event schemas activating and updating their weights most of the time. Relatedly, because event schemas in SEM-1.0 were trained to predict current scene vectors from either previous scene vectors or random vectors, the architecture's predictive power was compromised. We therefore modified SEM-2.0 so that both active and inactive schemas were provided with the previous scene vectors as input. To retain efficient processing, we parallelized the calculation of predictions from active and inactive schemas. These changes led to more even use of event schemas and reduced prediction error (Figs. S2 and S4).

Like SEM-1.0, SEM-2.0 performs inference over all schemas at every timestep, which is not biologically plausible. Gating mechanisms were added to create two model variants: uncertainty-SEM and pe-SEM. At each timestep, each of the two variants used their active RNN to make a prediction; then, prediction error and prediction uncertainty were computed. If prediction error (pe-SEM) or prediction uncertainty (uncertainty-SEM) exceeds the average prediction error or prediction uncertainty of the active RNN by a certain margin, the Bayesian inference process is triggered to determine the most probable RNN. Prediction error is operationalized as the Euclidean distance between the active RNN's prediction and the observed scene vector. There are two sources of uncertainties associated with an RNN's prediction: noise inherent in the data (aleatoric uncertainty) and the variance of the estimated RNN's weights (epistemic uncertainty) (24). We assume aleatoric uncertainty is constant for all data points (scene vectors) and conceptualize epistemic uncertainty as prediction uncertainty of the active event schema (RNN). In principle, epistemic uncertainty can be modeled by placing a prior distribution over an RNN's weights and then trying to estimate the posterior distribution of these weights given the observed scene vectors assigned to that RNN. This approach is computationally prohibitive in practice with large-scale datasets and especially with our architecture since we need to estimate posterior distributions of all RNNs’ weights. For RNNs, epistemic uncertainty can be approximated by randomly applying dropout before every weight layer and calculating the variance of predictions (Eq. 1) (32, 65). Specifically, at every timepoint *t*, we repeatedly generate predictions *S* times and estimate the variance of this prediction distribution.


(1) $$\begin{array}{rl} \mathrm{Uncertainty}(t) & =\frac{1}{S-1}\overset{S}{\underset{s=1}{\sum }}\;(v(s,\;t)-E[v(t)]{)}^{T}(v(s,\;t)-E[v(t)]);\\ & \;v\in R^{30},\;s,\;t\in D\\ E[v(t)] & =\frac{1}{S}\overset{S}{\underset{s=1}{\sum }}\;v(s,\;t) \end{array}$$

All code was implemented in Python, using the TensorFlow library for neural network implementation: https://github.com/mbezdek/extended-event-modeling and https://github.com/NguyenThanhTan/SEM2.

### Materials

Each of the three models was trained on the META stimulus set (25) which contains over 25 h of everyday activities, each about 10 min long. We used 128 performances out of 149 performances (Fig. S1). Performances were captured with a Kinect V2 device, which includes a video camera and a time-of-flight depth sensor (66), and two other video cameras. The Microsoft Kinect SDK was used to determine actors’ skeletal joint positions. Joint positions were adjusted, so the mid-spine joint was at the origin and rotated to align shoulder joints along the *Z*-axis. Features were smoothed with a rolling mean of seven frames. We calculated joint velocity, acceleration, and inter-hand distance metrics.

Semantic information about objects was captured as follows: Human annotators labeled object positions and identities with bounding boxes for a subset of frames at 10-s intervals. A tracking model (Siam region proposal network) was then used to track objects forward and backward between labeled frames (67). Trackers were dropped if confidence fell below a threshold, and the Hungarian algorithm matched tracks. Object appearance/disappearance features were binary, indicating frames where objects began or ceased being tracked. We embedded object names into 50-dimensional semantic vectors using the GloVe language model (27), averaged across objects in each frame, to create a feature set. We also calculated features for the three nearest objects to the actor's right hand, using a weighted average of vectors based on the inverse distance from the hand. In total, there were 102 object-related features: object appearances/disappearances, 50 features for all objects, and 50 features for the nearest objects to the actor's hand.

We performed principal component analysis to reduce the dimensionality of the feature vectors. Dimensionality reduction was performed separately on body motion and semantic features, to allow for modality-specific calculations of predictions and errors. The resulting set of features contained 30 dimensions (14 body motion dimensions, 13 semantic dimensions, 2 dimensions for object appearances and disappearances, and 1 dimension for the correlation of pixel luminance between successive video frames). This dimensionality reduction preserved 76% of the original variance of the full feature set.

### Training and evaluation regimen

To train and validate the models, we used a hold-out procedure to save computation time. The 128 activities were divided into a training set of 108 activities and a validation set of 20 activities. The training set was shuffled eight times to create eight sequences, each used for one simulation. In each simulation, each model variant watched one unique activity sequence, allowing them to learn from each activity only once, similar to human experience. Different instances of the same event type enabled the models to learn generalized event schemas. Each model's core architecture has two parts: a library of event schemas represented by RNN weight matrices and an approximate Bayesian inference module. Learning involved either creating new RNNs or adjusting weights of existing ones. At each timestep, only the currently active RNN processed the scene vector and updated its weights via backpropagation of prediction error. After every 10 training activities, models were evaluated using all validation activities with frozen RNN weights. Since each model ran eight simulations and was evaluated after every 10 activities, each model had eight data points per evaluation metric described below.

### Evaluation metrics

#### Prediction error

To measure how a model learns to predict throughout training, we calculated its prediction error for each pass through the validation set. The prediction error for each timestep is the Euclidean distance between the active model's prediction and the input scene vector at that timestep, and the summarized prediction error for the validation set is the average of the prediction errors calculated at all timesteps for all activities.


(2) $$\begin{array}{rl} & \mathrm{Validation}\;\mathrm{PE}\\ & =\frac{1}{\left|\mathrm{activities}\right|}\sum_{a\in \mathrm{activities}}\frac{1}{\left|\mathrm{timesteps}\right|}\sum_{t\in \mathrm{timestep}s_{a}}\sqrt[{2}]{{\left(v{\left(t\right)}_{\mathrm{predicted}}-v{\left(t\right)}_{\mathrm{input}}\right)}^{2}};\\ & \;v\in R^{30} \end{array}$$

### Scaled point-biserial correlation

To quantify models’ segmentation agreement with human segmentation, we computed scaled point-biserial correlations (68) between models’ event boundaries and human fine-grain segmentation. We treated the human fine-grain segmentation as the continuous variable and the models’ event boundaries as binary variables. To the extent that the models’ event boundaries correspond to peaks in human segmentation, the scaled point-biserial correlation between models and human segmentation will be high.

### Adjusted mutual information

To quantify models’ categorization agreement with human categorization, and how models’ event schemas generalize to actions performed by the same actor in different instances and by different actors at different locations, we computed the mutual information score (with adjustment to account for chance (37)) between the models’ event labels and human action labels. We treated script action labels as one clustering of input scene vectors, and each model's event labels as another clustering of input scene vectors. To the degree that the model categorizes input scene vectors in a human-like way and its event schemas generalize to instances of the same action, adjusted mutual information between two partitioning algorithms will be high (Fig. S8).

### Generating permutations

We wanted to assess the extent to which the correspondence of model boundaries and event labels with human boundaries and action categories could be due to the distribution of model event lengths. Recall that in order to appropriately compare the models’ segmentation to human segmentation, we tuned parameters so that the models produced event boundaries with the same median frequency as human participants. The models’ distributions of event lengths constrain when event boundaries could occur over the course of an activity. (For example, if the shortest event produced by a model was *k* seconds in duration, there could be no model event boundaries in the first or last *k* seconds of the activity.) To address this issue, we generated permutations from all three models (SEM-2.0, pe-SEM, and uncertainty-SEM). For each model, event labels for all validation activities were first concatenated. A permutation is generated by shuffling events (runs of event labels), thus preserving event lengths in the resulting permutation. The shuffling not only changes models’ event labels for particular input scene vectors but also changes models’ event boundaries. As a result, the shuffling procedure can be used for both segmentation and categorization tests. When we concatenate two validation activities, there is an interval between the onset of the last event in the first activity and the onset of the first event in the second activity. Because human event boundaries are less likely to fall into these intervals, and the models never placed boundaries in these intervals, we made sure permutations did not have boundaries within these intervals so that the models would not have an unfair advantage over permutations. Permutations were repeated one time for each model in each simulation, and because we ran eight simulations for each model, there were 48 permutations in total. Scaled point-biserial correlation, adjusted mutual information, purity, and coverage were computed for each permutation.

## Supplementary Material

pgae459_Supplementary_Data

## Data Availability

The META stimulus set is publicly available through its Open Science Foundation repository: https://osf.io/3embr/. All processing scripts are available at: https://github.com/mbezdek/extended-event-modeling and https://github.com/NguyenThanhTan/SEM2.

## Preprints

This manuscript was posted on a preprint: https://doi.org/10.31234/osf.io/pt6hx.
