# Supplementary material for: Modeling human activity comprehension at human scale: Prediction, segmentation, and categorization
Source: PNAS Nexus. 2024 Oct 11;3(10):pgae459. doi: 10.1093/pnasnexus/pgae459 (PMC11497596; doi:10.1093/pnasnexus/pgae459)
Supplement: pgae459_Supplementary_Data [file pgae459_supplementary_data.docx]

**
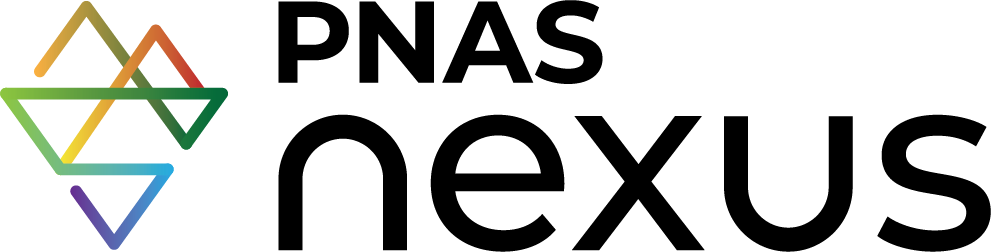
**

**Supplementary Information for**

Modeling human activity comprehension at human scale: Prediction, segmentation, and categorization

Tan T. Nguyen^1,^*^,^**, Matthew A. Bezdek^1,^*, Samuel J. Gershman^2^, Aaron F. Bobick^3^, Todd S. Braver^1^, Jeffrey M. Zacks^1,^**

^1^Department of Psychological and Brain Sciences, Washington University in St. Louis, St. Louis, MO, USA

^2^Department of Psychology and Center for Brain Science, Harvard University, Cambridge, MA, USA

^3^Computer Science and Engineering, Washington University in St. Louis, St. Louis, MO, USA

*These authors contributed equally to this work.

**Corresponding authors:

Tan T. Nguyen: [n.tan@wustl.edu](mailto:n.tan@wustl.edu) or

Jeffrey M. Zacks: [jzacks@wustl.edu](mailto:jzacks@wustl.edu)

**This PDF file includes:**

Supporting text

Figures S1 to S20

Tables S1 to S3

SI References

**Supplementary Information Text**

**Visualization of input features and SEM’s prediction**

We rendered input features, SEM-2.0’s prediction and segmentation for SEM versions with full input features, without semantic features, and without motion features in the directory “output_activities” stored in OSF: <https://osf.io/39qwz/?view_only=d186e6c784fa47f4a2da01ffcf08d850>. Description of these videos can be found in the same directory.

**META Stimulus Set**

The META stimulus set is comprised of over 25 hours of humans performing activities in naturalistic settings. Each activity was one of four different types of activities (making breakfast, exercising, cleaning a room, or bathroom grooming) and was performed by one of five different actors in a unique realistic room for each activity type, for a total of 15 environments. An algorithm generated scripts of six to seven actions for each activity, five to six actions sampled from a set of nine to 13 actions of a specific activity type, and one action sampled from a set of six actions that could appear in any activity type (see Fig. 5A for the sequences of actions for one activity type). The algorithm randomly selected actions from sets of sub-activity actions, with rules for excluding generated scripts with unrealistic combinations or orders of specific actions (for example, applying lotion before washing or shaving one’s face). The process was repeated so that for each combination of actor and activity type there were ten activity scripts with unique sequences of actions to perform. In total, there were 149 activities. Actors memorized and performed the actions for each activity consecutively with no breaks in the recording, and there was no dialogue or other actor present in any of the activities. Each performance was filmed with three synchronous color cameras and a time-of-flight depth sensor. For the set of activities used in this study, there was an average duration of 10 minutes and 22 seconds per activity. For more detailed information on the META stimulus set, please see the cited paper (*1*).

**Filtering thresholds for activity inclusion**

The Kinect body tracking produced large-scale errors for some of the activities (for example, in activities where the actor’s lower body was occluded, the Kinect mistook the actor’s upper body as the whole actor). To prevent activities with large tracking errors from having an undue influence on the model’s performance, we developed a filtering algorithm to select high-quality activities for training and validation. First, we calculated the range of all possible values for each derived body motion feature. The algorithm has two thresholds: 1) one threshold to mark bad features and 2) one threshold to filter bad activities. For each feature in each activity, the feature was considered good if y% of the values for that feature fell inside of the ninetieth percentile of the possible range, otherwise, it was marked. If more than x% of all features of the activity were marked, the activity was filtered. A grid search was used to determine values for x and y. See Fig. S1 for the proportion of activities that are left after applying each pair of x and y thresholds. The x-axis and y-axis in Fig. S1 correspond to threshold x and threshold y respectively, and the annotated values indicate the proportion of activities left. Because the models showed learning saturation after watching approximately 70 to 90 activities (Fig. 3), we can afford to apply stringent thresholds to maintain high quality without worrying about depleting the training dataset. As a result, we ended up with 128 activities out of the 149 activities in the META stimulus set (86%); an activity is selected if more than 80% of its features are considered good, and a feature is considered good if more than 80% of its value fall within the ninetieth percentile of the possible range.

**Modifications from the original SEM implementation**

Inspecting the performance of the original SEM implementation (SEM-1.0) (*2*) on the META stimulus set (*1*) revealed that it spawned many event schemas but used only a few of them to assign to input scene vectors. Three reasons led SEM-1.0 to this behavior: 1) The sticky Chinese Restaurant Process (sCRP) preferred highly frequent events, which could lead to a self-perpetuating circle, 2) new event schemas were initialized to random weights without any knowledge of the dataset, and 3) inactive RNNs were at a disadvantage because they had to make predictions from random input scene vectors.

To evaluate how evenly SEM-1.0 uses its event schemas, we computed entropy for the distribution of schema activations on either training activities or validation activities. Because the range of entropy depends on the number of elements (or the number of schemas), comparing entropy between two SEM-1.0 variants with different numbers of event schemas could be misleading. Entropy was normalized based on the maximum possible entropy (uniform distribution) for each particular number of schemas.

In the sCRP, there is a term in the prior over schemas that biases SEM-1.0 towards reusing event schemas that were activated frequently in the past, facilitating generalization across events. However, this *rich-get-richer* property led SEM-1.0 to create a small number of event schemas; the more these schemas were used, the more likely they would be used again since they were highly frequent events. Substituting the sCRP with the sticky uniform process (sUP) steered SEM-1.0 towards activating a larger set of event schemas, and activations were distributed more evenly for both training and validation activities. In the following figures, we will abbreviate SEM-1.0 as “SEM1.” In Fig. S2, the effect of the sUP on schema activation can be seen by comparing SEM1 and SEM1-StickyUniformProcessPrior.

SEM-1.0 initialized random weights to newly spawned event schemas, reducing the predictive power of these event schemas and consequently making these schemas less likely to be activated. Suppose that SEM-1.0 has one previously learned event schema for making a toast and one newly spawned event schema for making apple juice. Now, suppose the input scene vector is one where the actor is making tea. The previously learned event schema has general knowledge about what objects should be present and about general forms and movements of actors, whereas a newly spawned event schema (an RNN with random weights) does not. Therefore, that previously learned schema’s prediction—even if not perfect—will still be closer to the input scene vector than the prediction from the newly spawned event schema. Thus, previously learned event schemas are more likely to be activated than newly spawned event schemas due to random weight initializations. To address this, we created a modified version of SEM-1.0, in which a generic model was trained alongside the event schemas, and new event schemas were initialized to the generic model’s weights. The addition of the generic model and the removal of the advantage for highly frequent event schemas helped SEM use a much larger set of event schemas, and activate them much more evenly for both training and validation activities (SEM1 versus SEM1-GenericInit-StickyUniformProcessPrior in Fig. S2). Fig. S3 shows examples of distribution of schema activations for different versions of SEM.

At each timestep, SEM-1.0 computes the likelihood for each RNN given the input scene vector. In principle, each RNN should be fed scene vectors from previous timesteps and asked to predict the current scene vector, and the predicted scene vector would be compared to the input scene vector to compute the likelihood. However, this approach is computationally expensive as the number of event schemas in SEM-1.0 grows. In SEM-1.0 (*2*), the authors circumvented this challenge by feeding random vectors to RNNs instead of scene vectors from previous timesteps and asking RNNs to make predictions from these random vectors. These predictions were cached and used to compute likelihoods for RNNs that were not active at the previous timestep. That approach reduced computation time drastically and made the training much faster. However, this approach placed inactive RNNs at a disadvantage because the input scene vectors to these RNNs were not informative for predicting the current scene vector. Consequently, inactive RNNs were less likely to be selected and less likely to update their weights (as only the active RNN updates its weights at a specific timestep). As a result, some RNNs that are activated initially snowball and dominate other RNNs, getting activated most of the time. Furthermore, because these RNNs must be able to predict the next scene vector from either previous scene vectors or random vectors, their predictive power is compromised compared to the situation where they only need to predict the next scene vector from previous scene vectors. In SEM-2.0, in addition to the aforementioned changes, we fed previous scene vectors to all RNNs and parallelized RNNs’ predictions to alleviate the computational challenge. SEM-2.0 created a smaller number of RNNs to capture the data and more importantly activated RNNs more evenly for both training and validation activities (Fig. S2). In addition, prediction error was reduced (Fig. S4).

**Generalization across actors and environments**

We qualitatively evaluated how SEM-2.0’s event schemas generalize across actors in this dataset. For many of the ground-truth action labels, there were multiple action instances performed by the same actor in a different activity instance or performed by a different actor in a different environment. Thus, we were able to test whether across instances, actors, and environments, SEM-2.0 would assign the same event label to the same script action label. Fig. S5A shows a confusion matrix of the proportion of timepoints from each scripted action label instance in the validation set assigned to each SEM-2.0 schema for one trained instantiation of SEM-2.0. Several of SEM-2.0’s event schemas generalize across actors. For example, in this instantiation of SEM-2.0, schema 18 is the most frequently activated schema for the scripted action ‘wash face’ across instances, whereas schema 14 is the most frequently activated schema for ‘perform bicep curls’ across multiple actors. In general, SEM-2.0 generates separate sets of schemas for separate activity types and often over-generalizes to the activity type level. Fig. S5B shows the correspondence between schemas and action labels after shuffling the assignments of schemas to timepoints. Uncertainty-SEM and pe-SEM produced qualitatively similar results and are not shown here, for a quantitative comparison between the three models, see Fig. 5 in the main script.

**Purity and coverage comparing event schemas and judges’ action categories**

To quantify the models’ agreement with humans in terms of clustering, and understand how the models use schemas over training, we calculated purity and coverage for all validation activities. Across validation activities, we concatenated models’ event labels for each frame to form a single vector, and concatenated script action labels to form an aligned single vector. Purity and coverage were computed by comparing the two label vectors. Purity is the degree to which frames on which an event schema is active belong to a single script action label. The formula for purity, with |x| denoting the total length of x, is:

$$Purity= \sum_{schema\in Schemas} \frac{\max_{action\in Actions} \left| schema\cap action \right|}{\left| schema \right|}$$

*( 1 )*

Coverage is the degree to which frames assigned to a script action label have a single active event schema; the coverage formula is:

$$Coverage = \sum_{action\in Actions} \frac{\max_{schema\in Schemas} \left| action\cap schema \right|}{\left| action \right|}$$

*( 2 )*

Purity and coverage range from 0 to 1, where one is the best possible score. A schematic illustration of purity and coverage is shown in Fig. S6.

If SEM assigns unique event labels to each input scene vector, its purity will be 1, but its coverage will be close to 0. If SEM assigns a single event label to all input scene vectors, its coverage will be 1, but its purity will be close to 0. Thus, purity and coverage represent a tradeoff. To address this tradeoff, in the main manuscript we compared models on adjusted mutual information (see the section below) that harmonizes purity and coverage: both purity and coverage must be high for mutual information to be high. Below, we report purity and coverage for all models. We assessed how likely it was that the results could occur by chance by creating permuted event labels: we shuffled models’ events while preserving event lengths and calculated purity and average for shuffled event labels to derive a null distribution (see Methods). Fig. S7 shows purity and coverage for models’ event labels and permuted event labels for all validation activities across training.

As shown in Fig. S7, all models’ coverage scores are greater than expected by chance (two-sided t-tests, t-statistics are 23.56, 24.39, and 17.16, p-values are 1.8e-13, 2.4e-14, and 2.1e-11, and degree of freedom are all 62 for SEM-2.0, uncertainty-SEM, and pe-SEM, respectively) and decreases slightly across training, and SEM’s purity is bigger than expected by chance (two-sided t-tests, t-statistics are 13.07, 20.63, and 10.48, p-values are 6.6e-11, 9.9e-15, and 4.4e-10, and degree of freedom are all 62 for SEM-2.0, uncertainty-SEM, and pe-SEM, respectively) and increases across training. These trends indicate that early in training, all models have a relatively small number of generalist event schemas to accommodate the stimuli. Later in training, models’ event schemas become more specialized and correspond more to script action labels (see Fig. 7A). This effect seems to be more pronounced in uncertainty-SEM, where it ended up having the highest purity (two-sided t-test for SEM-2.0 vs. uncertainty-SEM: t-statistics=-4.80, p-value=4.1e-05, df=30; two-sided t-test for uncertainty-SEM vs. pe-SEM: t-statistics=10.36, p-value=4.1e-11, df=30) and lowest coverage (two-sided t-test for SEM-2.0 vs. uncertainty-SEM: t-statistics=9.89, p-value=1.4e-09, df=30; two-sided t-test for uncertainty-SEM vs. pe-SEM: t-statistics=-5.50, p-value=1.9e-05, df=30). SEM-2.0 had higher purity (two-sided t-test SEM-2.0 vs. pe-SEM: t-statistics=2.86, p-value=0.0078, df=30) and coverage (two-sided t-test SEM-2.0 vs. pe-SEM: t-statistics=4.50, p-value=0.00011, df=30) then pe-SEM did.

**Mutual Information**

Given a stimulus set, *Corpus*, with N input scene vectors (the total number of input scene vectors across all validation activities), and two partitions of Corpus, namely *Schemas* (e.g., SEM-2.0’s categorization) and *Actions* (humans’ categorization):

$$Corpus = \left\{ v_{1}, v_{2}, \ldots, v_{n} \right\}; v_{i} \in R^{30}$$

*( 3 )*

$$Schemas = \left\{ schema_{1}, schema_{2}, \ldots, schema_{n} \right\}; schema_{i} = \left\{ v_{i}, v_{j}, \ldots, v_{k} \right\}$$

*( 4 )*

$$Actions = \left\{ action_{1}, action_{2}, \ldots, action_{m} \right\}; action_{i} = \left\{ v_{i^{'}}, v_{j^{'}}, \ldots, v_{k^{'}} \right\}$$

*( 5 )*

The mutual information score between Schemas and Actions is computed as:

$I\left( Schemas; Actions \right) =\sum_{y\in Schemas} \sum_{x\in Actions} P\left( schema, action \right)\log\left( \frac{P\left( schema, action \right)}{P\left( schema \right)P\left( action \right)} \right)$

*( 6 )*

Concretely, equation 6 is a summation over all schema-action pairs. An example of a schema-action pair is shown in Fig. S8, and the calculation for that pair is below:

$$P\left( Event 4; Jumping Jacks \right)\log\left( \frac{P\left( Event 4; Jumping Jacks \right)}{P\left( Event 4 \right)P\left( Jumping Jacks \right)} \right)$$

*( 7 )*

Equation 7 was repeated and summed for all pairs of SEM-2.0 event schemas and scripted action labels to derive a mutual information score (equation 6).

For each pair of Schema_i_ (e.g. SEM-2.0 event schema “4,” meaning the 4^th^ schema created by SEM-2.0 during training) and Action_j_ (e.g. script action label “jumping jack”), P(event 4, jumping jack) is the probability that an input scene vector belongs to both clusters event 4 and jumping jack. If cluster event 4 corresponds with cluster jumping jack (a large number of timesteps are labeled as both SEM-2.0 event schema 4 and script action label jumping jack), the ratio within the logarithmic function will be high and the term for this pair will also be high. The mutual information score can also inform us about each model’s ability to generalize across actors and activities. A given action (e.g., jumping jack) can be performed by different actors in different environments. If the model is able to generalize across actors and environments, it should assign the same event label (e.g., event 4) to those input scene vectors. In that case, the adjusted mutual information score will be high. In contrast, if the model assigns different event labels to the same action performed by a different actor in a different room (event 4 to actor A jumping jack and event 20 to actor B jumping jack), the score will be low. To the degree that the models categorize input scene vectors in a human-like way, and their event schemas generalize to instances of the same action, adjusted mutual information scores will be high.

**Hyper-parameter search**

The goal of hyper-parameter tuning for SEM-2.0 was to achieve low prediction error on validation activities while ensuring that the model segmented events with the same granularity as human fine segmentation, to allow us to compare the timing of the models’ segmentation with that of the humans’. To select appropriate hyperparameters for SEM-2.0, we first performed a grid search for three parameters to minimize validation prediction error: stickiness, concentration, and learning rate. Stickiness could take one of the values in (1e-1, 1e0), concentration could take one of the values in (1e4, 1e5), and learning rate could take one of the values in (1e-2, 1e-3, 1e-4). Together, the three hyper-parameters form 12 sets of hyper-parameters (2x2x3). For each set of the three hyperparameters (e.g., stickiness=1e0, concentration=1e4, and learning rate=1e-2), six simulations with different random seeds were run. Those simulations from 12 hyper-parameter sets were pitted against each other to see which set of hyperparameters reduces prediction error the most. To evaluate the influence of each hyperparameter on prediction error, for each hyperparameter, simulations with the same value for that hyperparameter were collapsed and compared against simulations with a different value for that hyperparameter. Adjusting the learning rate had a large effect on prediction error, whereas adjusting stickiness and concentration did not noticeably affect prediction error (Fig. S9). We picked learning rate=1e-3 because models with that learning rate had the lowest validation prediction error. After selecting the appropriate learning rate, stickiness and concentration were then tuned so that the resulting SEM-2.0 simulations would have a similar median number of boundaries to that of human fine-grain segmentation, whose median number of boundaries per validation activity was 23 (Fig. S10). Stickiness was searched in three values (1e-1, 1e0, 1e1) and concentration was searched in three values (1e5, 1e6, 1e7), forming 9 sets (3x3) of hyper-parameters. The nine hyper-parameters sets resulted in SEM-2.0 simulations with similar prediction errors. We picked the configuration with the stickiness of 1e5 and concentration of 1e-1 since the median number of boundaries of SEM-2.0’s simulations with this combination was 24, closest to human’s median number of boundaries (Fig. S10). Note that because SEM-2.0 showed flurries of boundaries whereas human segmenters did not, comparing the raw number of boundaries between SEM-2.0 and humans could be misleading. To factor in these flurries, we performed the following adjustment to SEM-2.0’s event boundaries before counting them: if two or more boundaries were closer than two seconds, only one boundary was retained.

SEM-2.0 has a number of other hyperparameters that are not of theoretical interest. We set hyperparameters that control SEM’s sensitivity to error according to stimulus statistics, as suggested in SEM-1.0 (*2*); see table S1. We retained the previous hyperparameters for the Adam optimization algorithm and RNN architecture; see Table S2.

As in the case with SEM-2.0, the goal of hyper-parameter fine-tuning for uncertainty-SEM and pe-SEM was to achieve low validation prediction error while ensuring that the models segmented events with the same granularity as human fine-grain segmentation. As observed for SEM-2.0, learning rate 1e-3 had the lowest validation prediction error and we heuristically picked this learning rate to start fine-tuning uncertainty-SEM and pe-SEM. Note that with the new triggering mechanisms in these two variants, we introduced two new hyper-parameters: *prediction error threshold* and *prediction uncertainty threshold*. At timestep t, if prediction error is bigger than the average prediction error of the current event plus prediction error threshold, pe-SEM triggers the full inference process. The same mechanism applies to uncertainty-SEM with prediction uncertainty. The number of triggers depends on prediction error threshold and prediction uncertainty threshold. Not all triggers induced by prediction error or prediction uncertainty resulted in event model transitions because the event model transitions depend on prior probabilities (influenced by concentration and stickiness hyper-parameters) and likelihoods (influenced by the noise variance and degree of freedom hyper-parameters). Because we are interested in whether prediction error or prediction uncertainty is a better candidate for controlling event model transitions, we wanted most triggers to result in event model transitions. In other words, we adjusted hyper-parameters so that the ratio of the number of transitions to the number of triggers was higher than 0.7/ (We chose 0.7 as a quantitative instantiation of “most.”) For uncertainty-SEM and pe-SEM to segment events in the range of human fine-grain segmentation, and with the ratio of the number of boundaries to the number of triggers higher than 0.7, we first fine-tuned prediction error threshold and prediction uncertainty threshold such that the median number of triggers was close to 23/0.7~33. We then performed stickiness and concentration hyper-parameter search so that uncertainty-SEM and pe-SEM segment events at the same granularity with human fine-grain segmentation. At the end of training (>1000 minutes), uncertainty-SEM’s median number of triggers and boundaries were 31 and 25 respectively, and pe-SEM’s median number of triggers and boundaries were 32 and 23 respectively. After tuning the thresholds and concentration and stickiness, we then varied the learning rate for uncertainty-SEM and pe-SEM (1e-2, 1e-3, 1e-4) to verify the heuristic. Models with learning rate 1e-3 resulted in the lowest validation prediction error (data not included). The resulting hyper-parameters are in Table S3.

**Training and testing input-deprived models**

In the analyses below, we test the ability of uncertainty-SEM to learn predictive representations with limited input. We focused on uncertainty-SEM because it had the strongest correspondence with human segmentation and categorization.

Recall that the scene vectors presented to the model included two sub-vectors, one representing biological motion features and the other representing semantics of objects in the environment that could be interacted with. We trained two versions of uncertainty-SEM that generated predictions for the full scene vector but withheld from the input either the body movement features (*motion-deprived model*) or the semantic features (*semantics-deprived model*). The two models still had to make predictions for all features: 30-dimensional output scene vectors, including both semantics and motion features. We evaluated deprived models on three metrics: prediction error, scaled point-biserial correlation, and adjusted mutual information.

**Models deprived of some input features produce higher prediction errors**

For both the motion-deprived model and the semantics-deprived model, with a subset of input features, uncertainty-SEM was able to learn to predict scene vectors and to reduce prediction error over time, though prediction error remained higher than in the model that received the full set of input features (Fig. S11).

**The segmentation of full uncertainty-SEM corresponds more closely to human segmentation than does either of the two deprived versions**

We also compared segmentation agreement between models and humans for the motion-deprived model and the semantics-deprived model. We again used a permutation test to generate a null distribution for each type of model, and combined these null distributions into a single null distribution. All models performed better than change (two-sided t-tests, t-statistics are 35.95, 26.15, 32.43, and p-values are 4.80e-31, 1.2e-17, 5.4e-22, and degree of freedoms are all 62 for uncertainty-SEM, semantics-deprived, and motion-deprived respectively; Fig. S13). Uncertainty-SEM’s segmentation agreement with humans was higher than the semantics-deprived model (two-sided t-test: t-statistics=-2.66, p-value=0.012, df=30) and the motion-deprived-model (two-sided t-test: t-statistics=-4.44, p-value=0.0001, df=30). These results suggest that semantic information and motion information capture unique variance of human segmentation, and uncertainty-SEM was able to exploit the two complementary features to segment events. Examples of uncertainty-SEM segmentation and deprived models’ segmentation are in Fig. S12.

**Deprived model schemas show correspondence with human action categories**

To test if the schemas formed by deprived models align with human action categories, we computed adjusted mutual information between human action labels and the motion-deprived and semantics-deprived models. Uncertainty-SEM with full input features had higher adjusted mutual information scores than the motion-deprived model (two-sided t-test: t-statistics=-4.44, p-value=1.01e-04, df=30) and semantics-deprived model (two-sided t-test: t-statistics=-2.61, p-value=1.36e-02, df=30) did, and all models performed better than chance (two-sided t-tests, t-statistics are 3167, 33.29, 36.41, and p-value are 5.42e-40, 1.71e-44, 3.95e-42, and degree of freedom are all 62 for uncertainty-SEM, semantics-deprived, and motion-deprived models, respectively; Fig. S14). These results suggest that semantic information and motion information capture unique variance of human categorization, and uncertainty-SEM was able to exploit the two complementary features to categorize events.

**Generic models show signs of interference**

To assess the impact of event knowledge partitioning, we created a *generic model*, composed of a single RNN that predicted the incoming scene vector from the last input scene vectors, and compared its mean prediction error to SEM-2.0’s mean prediction error. This model used the same parameters as SEM-2.0 for its one event schema, but could not switch or spawn new event schemas. SEM-2.0’s library of RNNs adds a larger number of parameters above the generic model’s but the same amount of weight updating (learning) on each time step; the exact number of weights depends on the number of event schemas created. To address this issue, we also created variations of the generic model with double and triple the number of units in the hidden layer. This increases the number of weights to be more comparable with the number of weights in SEM-2.0—but gives the generic model a large advantage in the amount of weight updating it experiences. Specifically, whereas only the active model in SEM-2.0 updates its weights, the original and expanded generic models update all their weights on each time step, which increases the amount of weight updating 2.5 times and 4 times, respectively.

Whereas the generic models must update all their weights with each new input scene vector, the schema weight updating mechanism in SEM-2.0 is able to silo data from a newly encountered event without compromising the integrity of the other event schemas in its library, which reduced interference. To illustrate the interference of new learning with previous learning in SEM-2.0 and generic models, we plotted average prediction error and prediction error for individual validation activities over training. As shown in Fig. S15 (top panels, B-D), the fluctuating lines (each line corresponds to a random initialization of weights and training activity order) indicate that the generic models’ average prediction error for validation activities sometimes increased as they saw more training activities, suggesting that the new learning from recent training activities had interfered with previously acquired learning that was beneficial to predict validation activities. Fig. S15 (bottom panels) shows prediction errors for all validation activities across training for the “red” simulation. Qualitatively, the generic model shows catastrophic interference at many points across training (e.g., around minute 510-th or minute 800-th), with almost all validation activities’ prediction errors increasing. The same pattern can be observed in the generic models with double and triple numbers of hidden units.

To quantify the interference effect, we calculated the first derivative of prediction error for all validation activities across all simulations. For each validation activity in each simulation, we computed the logarithm of the ratio of positive first derivatives to negative first derivatives (log-ratio), separately for SEM-2.0 and generic models. Independent sample t-tests show that the log-ratios of generic models were significantly higher than the log-ratio of SEM-2.0 (t-statistic and p-value for generic model, generic model with double and triple hidden units are: t-statistic=-5.78, p-value=4.43e-09; t-statistic=-3.51, p-value=2.32e-4; statistic=-3.57, p-value=1.8e-5).

**Equating stickiness and concentration for uncertainty-SEM and pe-SEM**

The concentration and stickiness hyperparameters required to cause uncertainty-SEM and pe-SEM to segment at a similar grain to human fine segmentation differ. One might ask whether the observed differences between the two variants in segmentation agreement with humans and in event categorization could be due to those hyperparameter differences. To address this, we compared uncertainty-SEM with a version of pe-SEM whose stickiness and concentration were the same as uncertainty-SEM (called parameter-matched-pe-SEM). Conversely, we compared pe-SEM with a version of uncertainty-SEM whose stickiness and concentration were the same as pe-SEM (called parameter-match-uncertainty-SEM).

At the end of training (>900 minutes), uncertainty-SEM had a significantly higher scaled point biserial correlation with human event boundaries than parameter-match-pe-SEM did (two-sided t-test: t-statistics=8.67, p-value=7.4e-08, df=30; Fig. S16). Uncertainty-SEM also had significantly higher adjusted mutual information with human action labels than parameter-match-pe-SEM did (two-sided t-test: t-statistics=-7.43, p-value=4.27e-08, df=30; Fig. S17). Parameter-match-uncertainty-SEM had significantly higher scaled point biserial correlation with human event boundaries than pe-SEM (two-sided t-test: t-statistics=-15.85, p-value=5.2e-12, df=30, Fig. S18). Parameter-match-uncertainty-SEM also had significantly higher adjusted mutual information with human action labels than pe-SEM did (two-sided t-test: t-statistics=3.09, p-value=4.25e-03, df=30; Fig. S19). These results robustly indicate that a model variant that triggers event schema inference based on prediction uncertainty segmented events and categorized scenes in a more human-like manner than a model variant that triggers event schema inference based on prediction error.

**The generation and use of event schemas**

A notable aspect of the models is how they form new event schemas and use event schemas over the course of training. Fig. S20A shows the number of spawned event schemas and Fig. S20B shows the number of event schemas that were active for more than 300 s (out of 12852s) in the validation dataset, over the course of training. Early in training, when the models haven’t learned much, they proliferate new event schemas to capture the statistics of the stimuli. In the middle and late stages of training, all models’ number of schemas seem to asymptote, suggesting that the models start to reuse their event schemas to accommodate novel stimuli while using these novel stimuli to update the weights of their existing event schemas. Even though the three models spawn similar numbers of event schemas (40 to 50 by the end of training), uncertainty-SEM uses event schemas more evenly: more of its schemas are selected for a significant duration during the test set (more than 300 seconds), whereas pe-SEM has more schemas that are created but rarely activated.


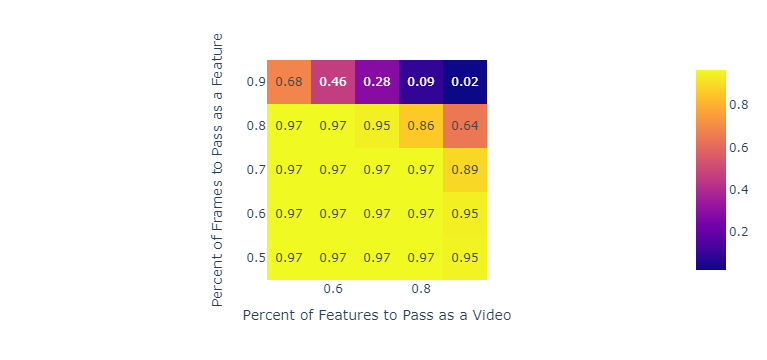


Fig. S1. The proportion of activities passing the filtering algorithm with different combinations of two thresholds. The Y-axis represents the proportion of frames falling within the ninetieth percentile so that a feature can be marked as good. The X-axis represents the proportion of good features so that the video can be used for training and testing. Each annotated number is the proportion of activities passing the filtering algorithm with each combination of thresholds.


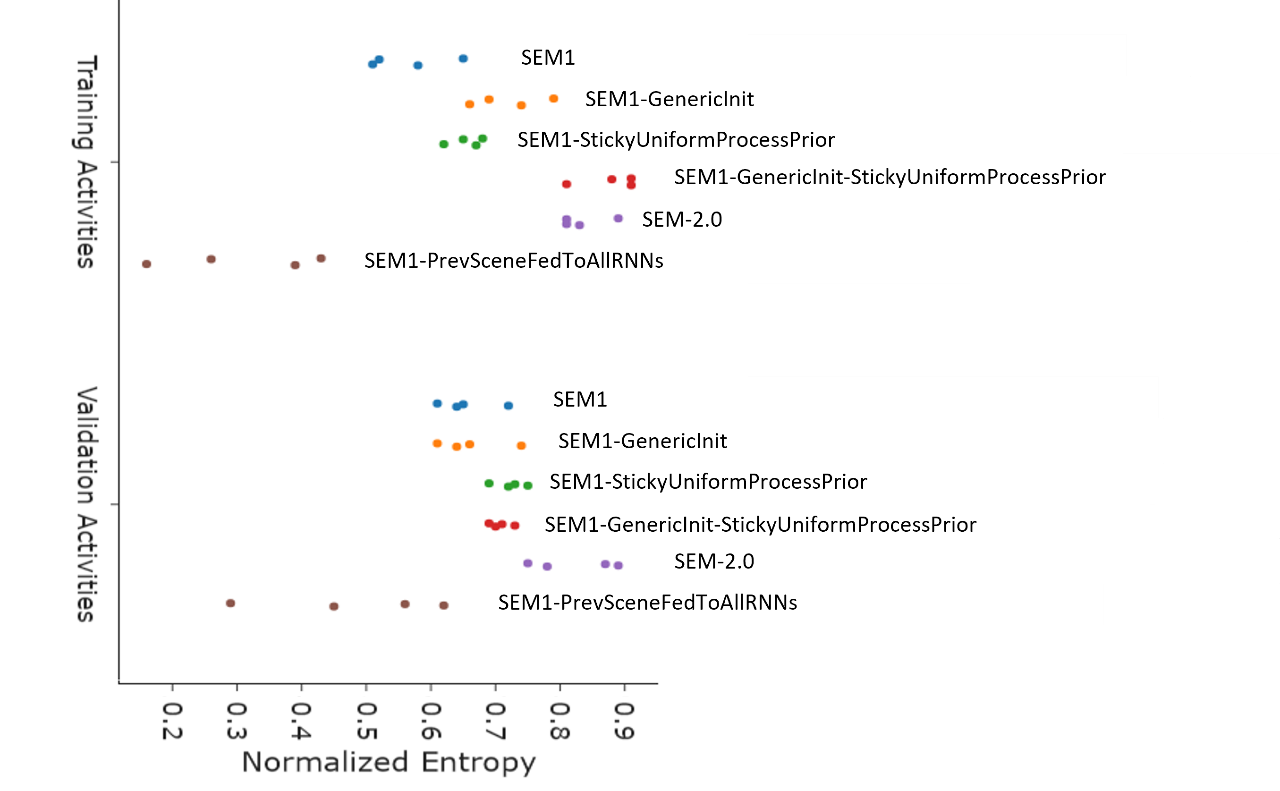


Fig. S2. Effects of modifications made in SEM-2.0 on the entropy of the distribution of event schema activations. Normalized entropy is the degree to which the number of frames each schema is activated is distributed equally across a large number of schemas during validation. Each SEM version was simulated with four different random initializations. SEM1 is original SEM (SEM-1.0). SEM1-GenericInit is the original SEM with one modification: initializing new RNNs to the generic model’s weights. SEM1-StickyUniformProcessPrior is the original SEM with one modification: using sUP instead of sCRP. SEM1-GenericInit-StickyUniformProcessPrior is the original SEM with the two modifications above. SEM1-PrevSceneFedToAllRNNs is the original with one modification: feeding the previous scene vectors to all RNNs instead of only active RNNs. SEM-2.0 is the version with three modifications. Initializing new RNNs to the generic model’s weights alone improved schema activations for training activities, and using the sUP alone improved schema activations for both training and validation activities. In contrast, feeding previous scene vectors alone hurts schema activations. The combination of three modifications increased the entropy of schema activations by a large degree on both training and validation activities.

**
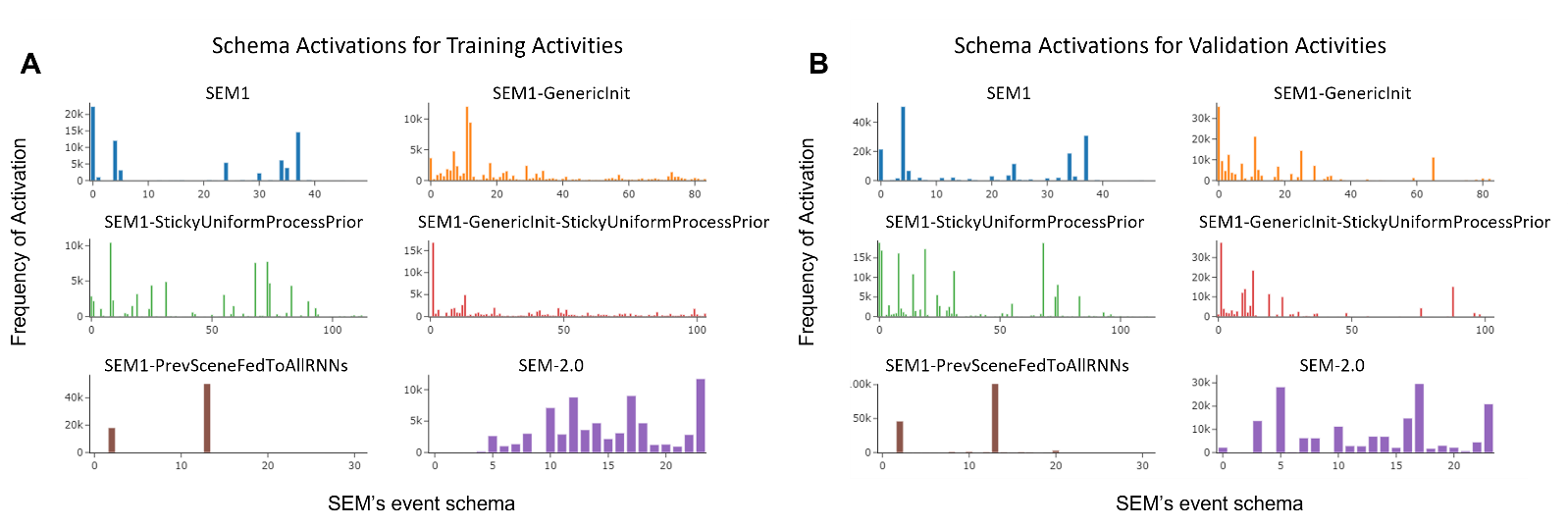
**

Fig. S3. Examples of schema activations for different versions of SEM for training (A) and validation (B) activities. All versions were simulated with the same random initialization. The x-axis indicates event schema number in the order spawned, and the y-axis depicts the number of timesteps on which the schema was active. For this particular random initialization, adding the generic model or using the sUP both made SEM activate schemas more evenly. In contrast, feeding previous scene vectors alone made SEM use only a few schemas. The combination of three modifications made SEM-2.0 create a smaller number of schemas and activate schemas much more evenly for both training and validation activities.


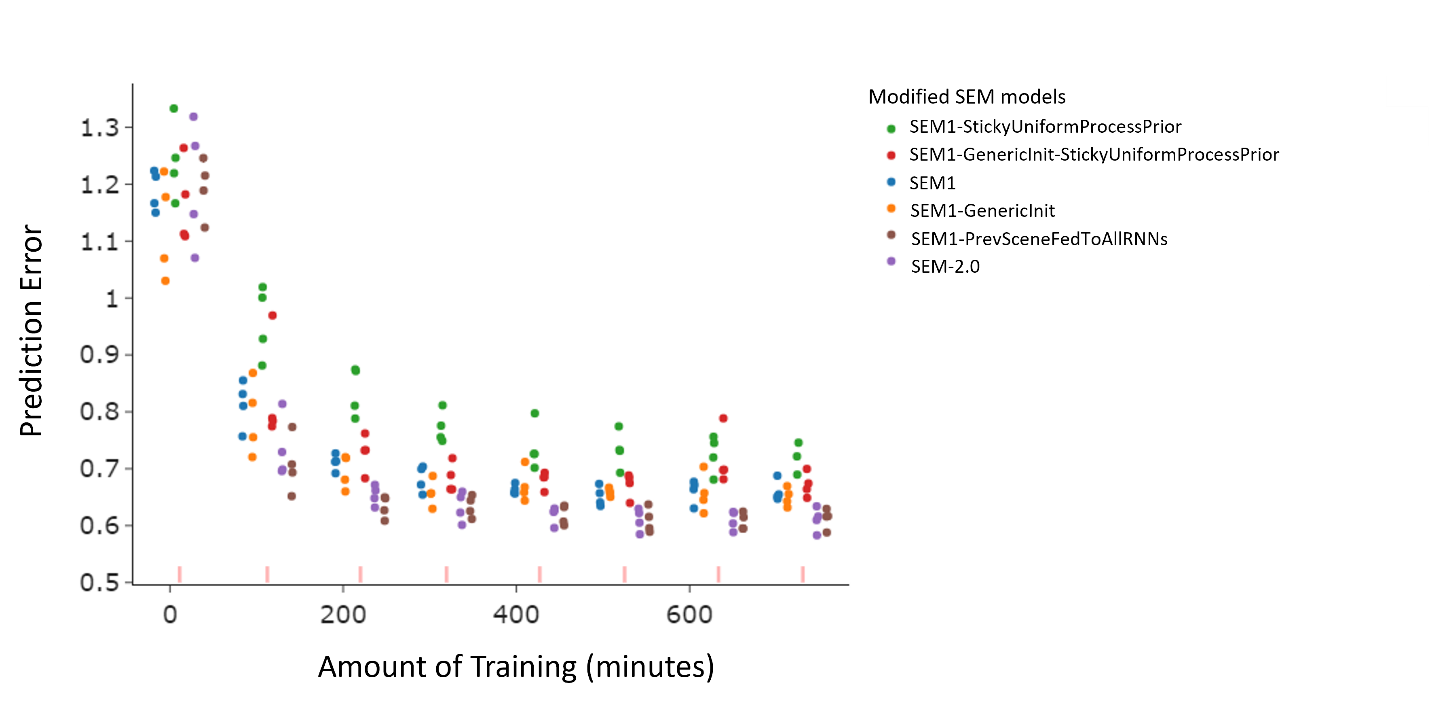


Fig. S4. The effect on prediction error of feeding previous scene vectors to RNNs. Using the sticky Uniform Process prior (SEM1-StickyUniformProcessPrior) increased prediction error while adding the generic model (SEM1-GenericInit) decreased prediction error a little bit. Points are jittered in the x dimension to reduce overlap; red ticks mark the sampled points in training. Adding previous scene vectors to all RNNs reduced prediction error (SEM1-PrevSceneFedToAllRNNs and SEM-2.0 had lower prediction errors than SEM-1.0’s).


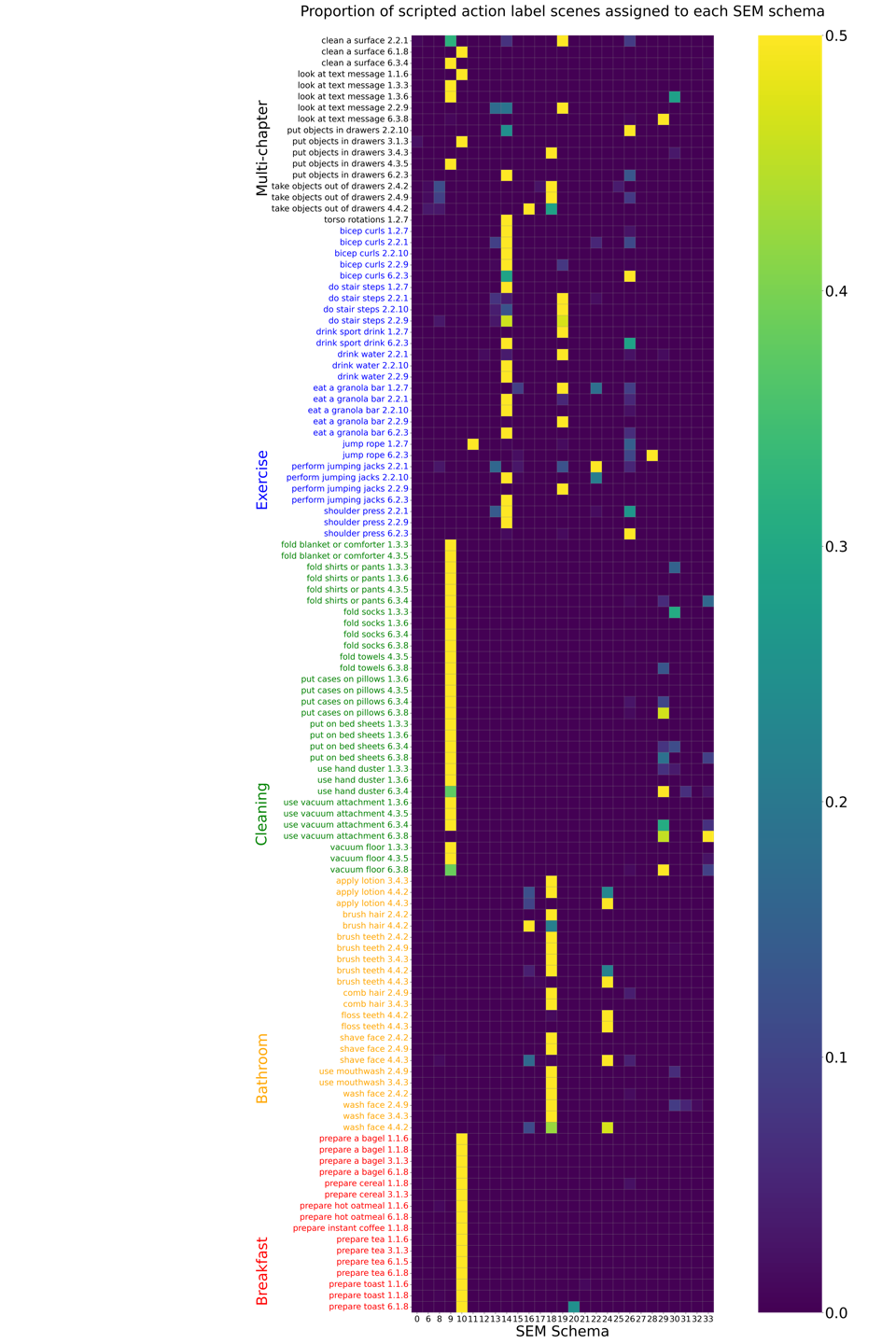

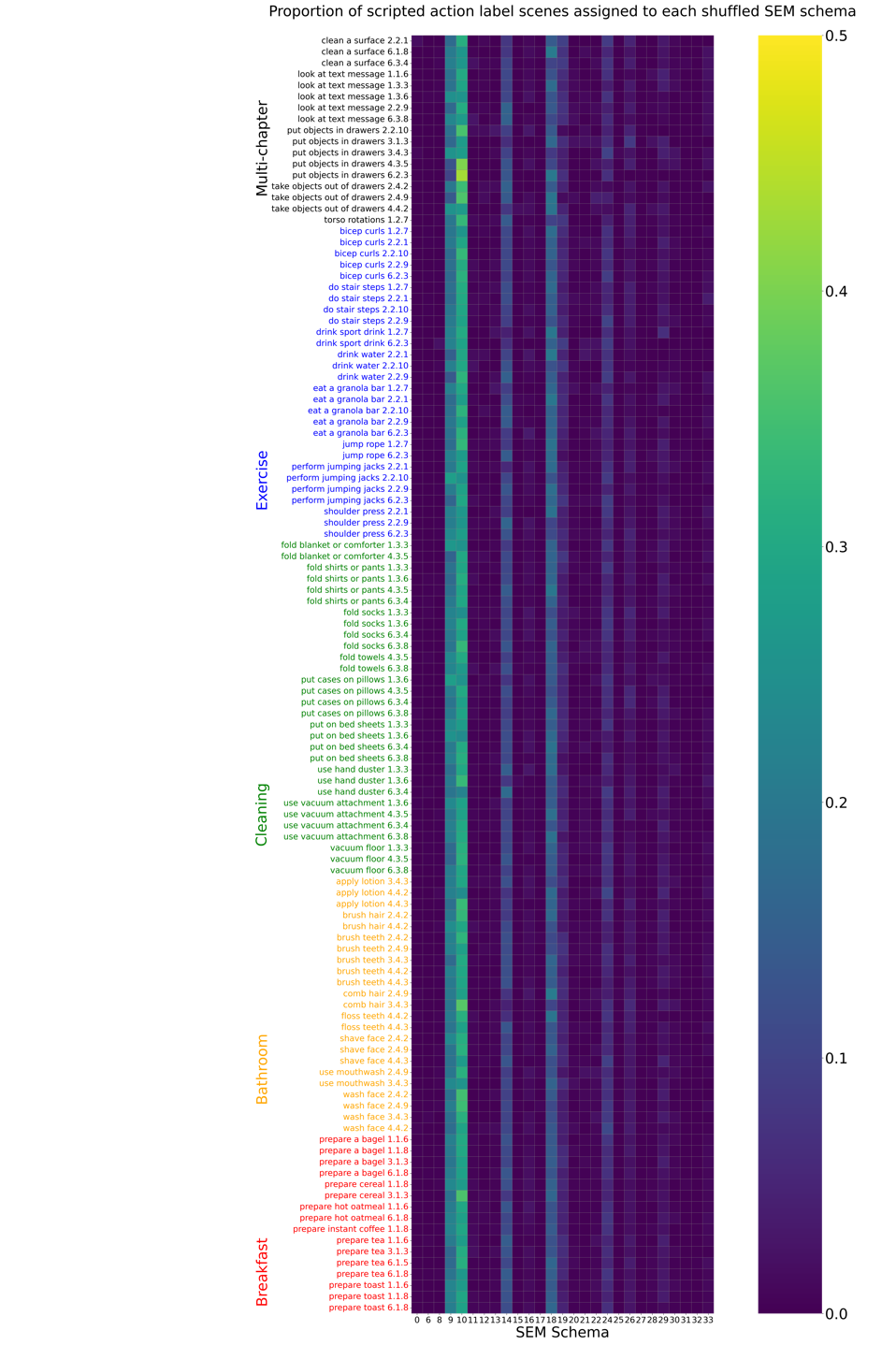


**B**

**A**

Fig. S5. Confusion matrix of the proportion of timepoints from each scripted action label instance in the validation set assigned to each SEM-2.0 schema, for (A) one initialization of SEM-2.0, and (B) the confusion matrix after randomly shuffling the assignment of SEM-2.0 schemas to timepoints. Scripted action labels are ordered by the type of activity they appeared in, with breakfast activity actions in red, bathroom activity actions in orange, cleaning activity actions in green, exercise activity actions in blue, and multi-activity actions in black. SEM-2.0 schemas are arranged in the order that they were spawned. Timepoints without a scripted action label and scripted action labels with just a single instance in the validation set are omitted. Several schemas generalize across instances, actors, and environments and some schemas over-generalize to entire activity types (e.g., schema 10 is recruited for almost all breakfast actions). After shuffling the SEM-2.0 schemas, there are visible differences in the relative frequency of schemas but no clustering according to action or activity type.


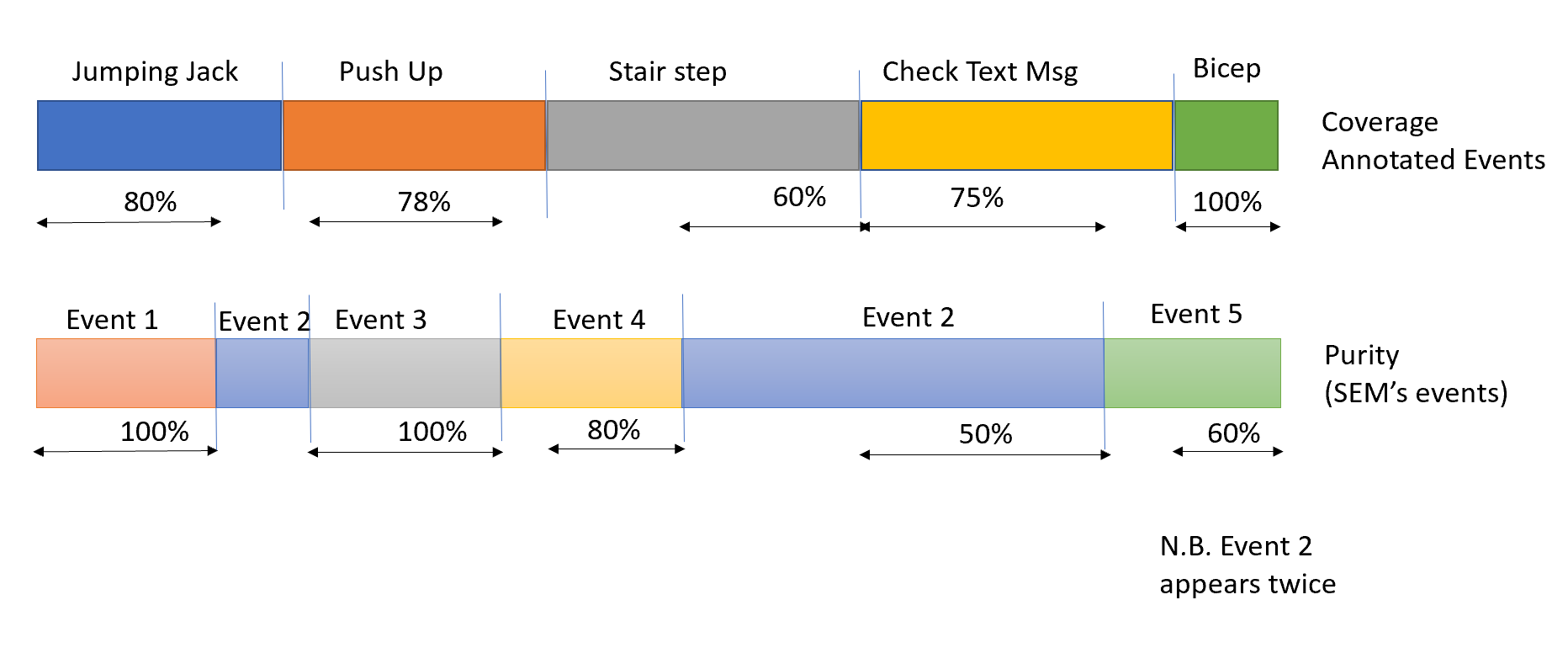


Fig. S6. A schematic illustration of purity and coverage for SEM’s event schemas and script actions. Percentages under action labels are their coverage, how much of that action is covered by a single event schema. Percentages under event schemas are their purity, the largest portion of the event schema matched with a single action label.


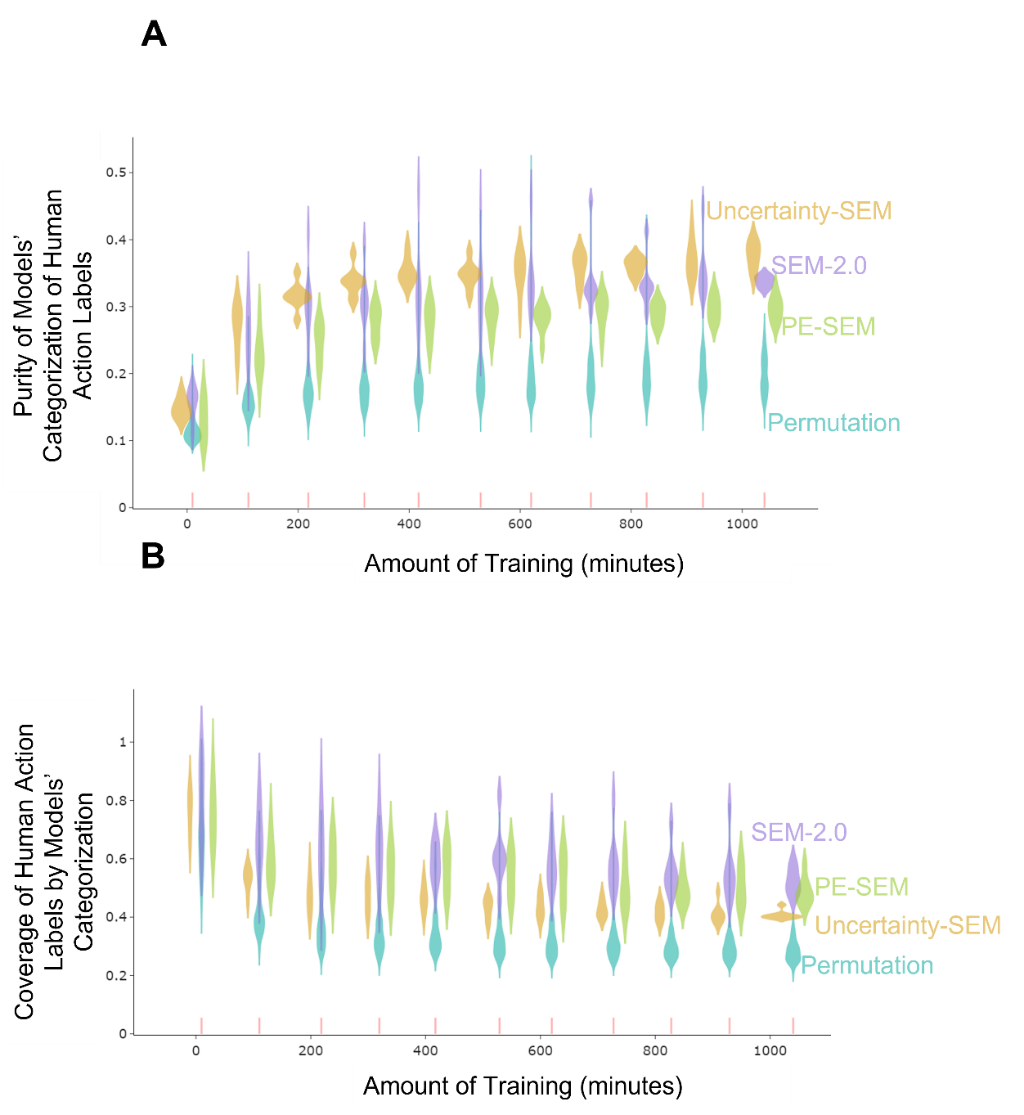


Fig. S7. Purity (A) and coverage (B) for multiple simulations of three models, compared against permutations across training. Permutations were performed such that event lengths are preserved. The plotted distributions are jittered in the x dimension to reduce overlap; red ticks mark true sampled timepoints. All models’ purity and coverage are bigger than expected by chance, and they show the tendency of activating more specialized event schemas as training progresses (purity increases over training while coverage decreases over training). This tendency is most pronounced in uncertainty-SEM.


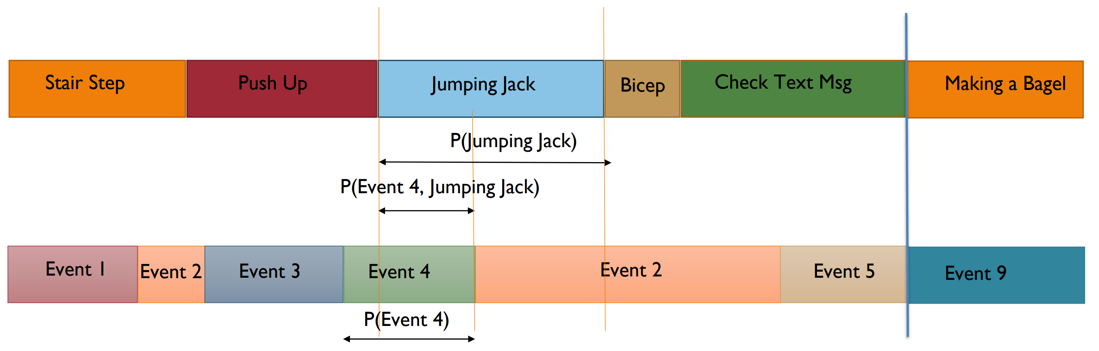


Fig. S8. Conceptual example of the calculation of mutual information. Top: a sequence of human actions. Orange vertical line indicates the start of one action and the end of another action. Blue vertical line indicates the transition between two activities (since all validation activities were concatenated to compute mutual information score). P(Jumping Jack) and P(Event 4) are marginal probabilities of jumping jack scenes and scenes that were assigned to SEM’s event 4. P(Event 4, Jumping Jack) is the joint probability of scenes assigned to event 4 and jumping jack. In this illustration, the result of equation 7 will be high since there is a high correspondence between event 4 and jumping jack.


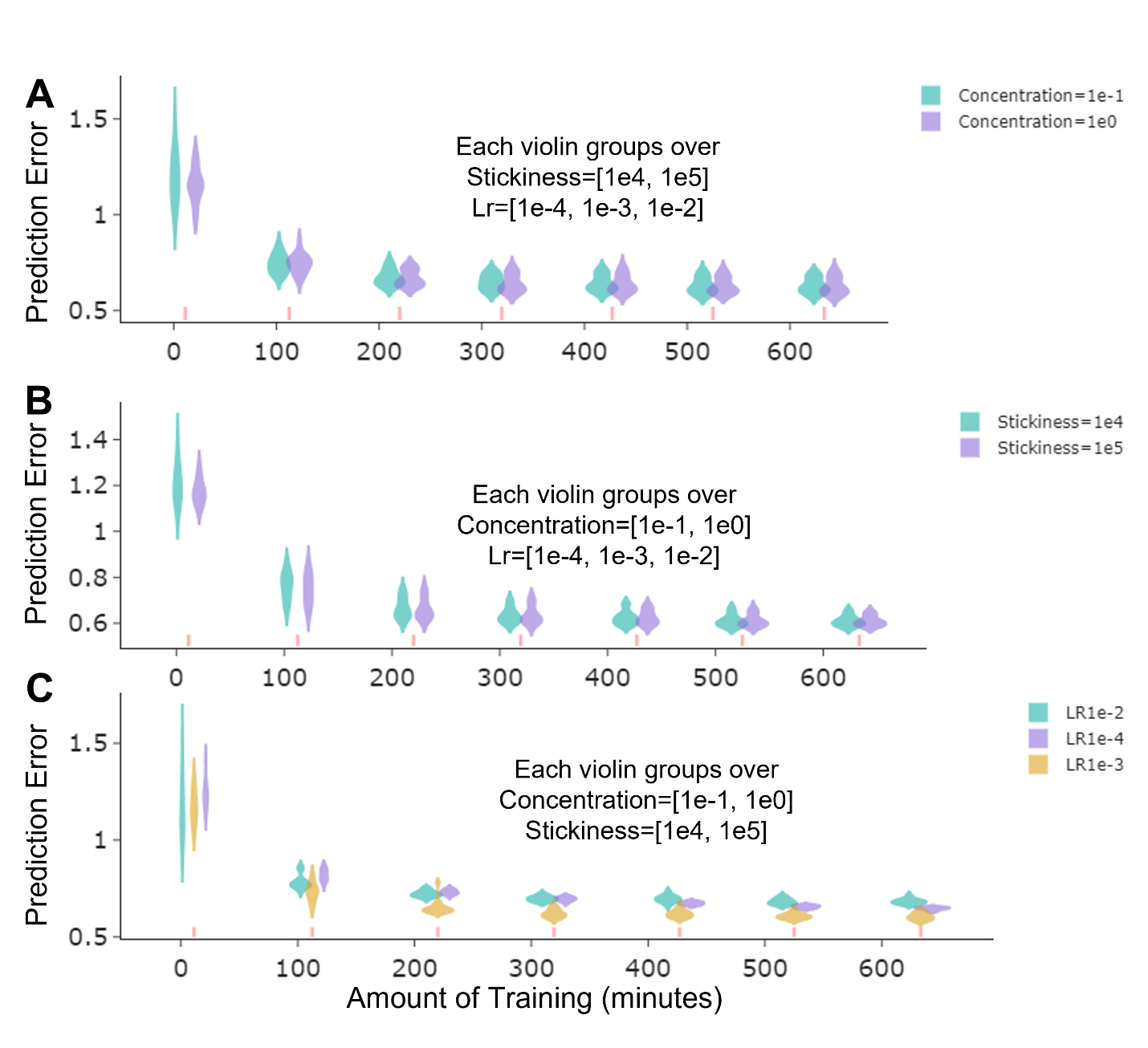


Fig. S9. The relative influence of hyperparameters on prediction error. A violin plot is shown for all simulations at each level of the given hyper-parameter (grouping all levels of the other two hyper-parameters). The plotted distributions are jittered in the x dimension to reduce overlap; red ticks mark true sampled timepoints. Changing stickiness or concentration didn’t affect prediction error much (A and B panels). However, changing learning rate affected prediction error significantly (C panel), and simulations with learning rate of 1e-3 performed best.


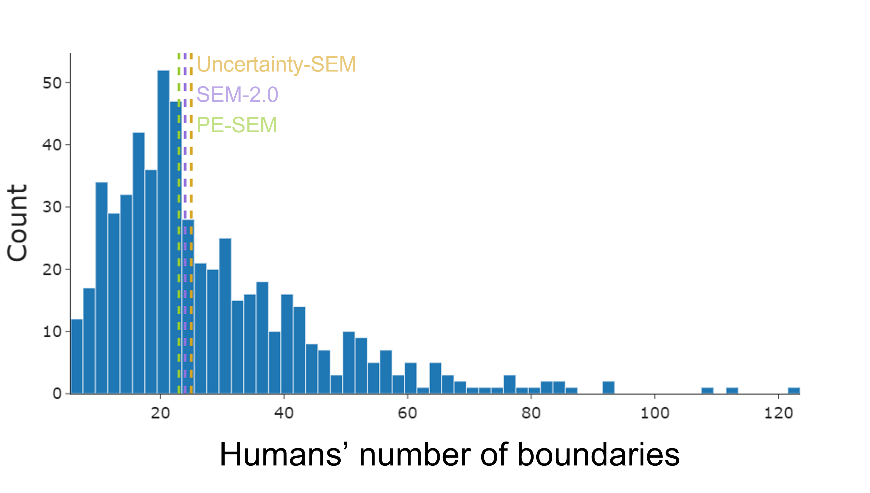


Fig. S10. Three models’ medians of adjusted number of boundaries and human boundaries. Histogram is the distribution of number of fine-grain boundaries identified by humans. Purple line, goldenrod line, and green line are the medians of adjusted number of boundaries for validation activities identified by SEM-2.0, uncertainty-SEM, and pe-SEM respectively.


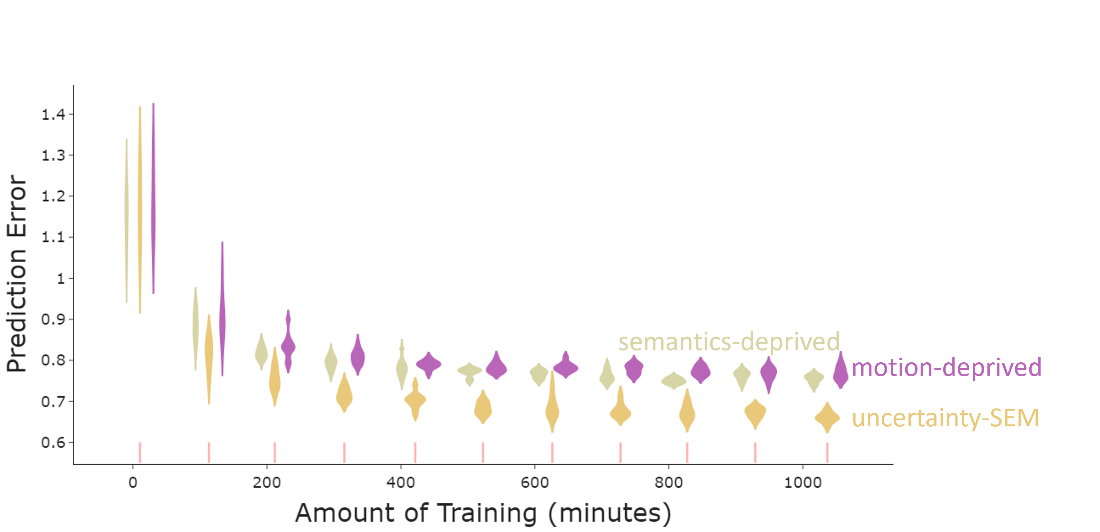


Fig. S11. Input-deprived versions of uncertainty-SEM produce higher error than uncertainty-SEM. For each uncertainty-SEM version, there were multiple simulations with different weight initializations and training orders. Each violin plot is a distribution of prediction errors for these simulations. The plotted distributions are jittered in the x dimension to reduce overlap; red ticks mark true sampled timepoints.


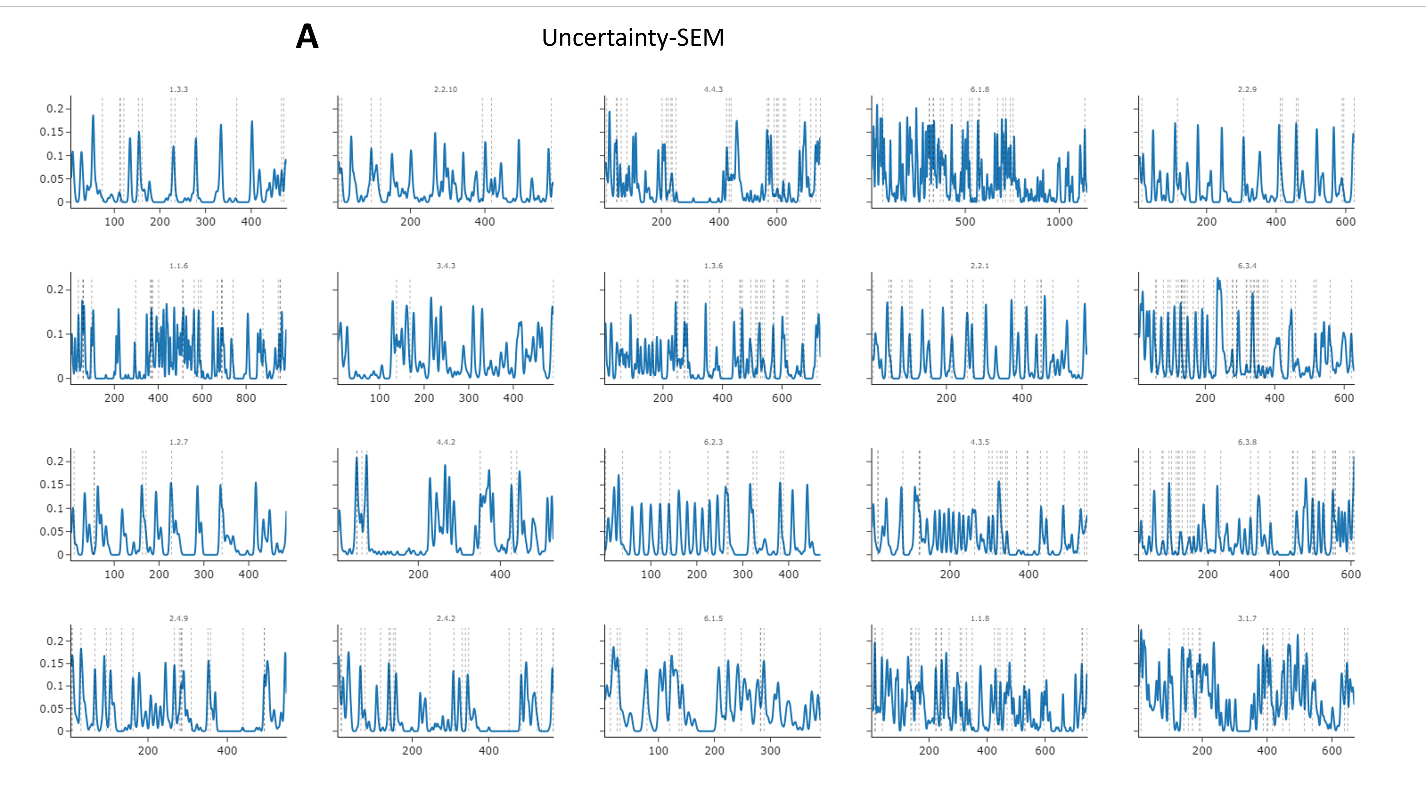


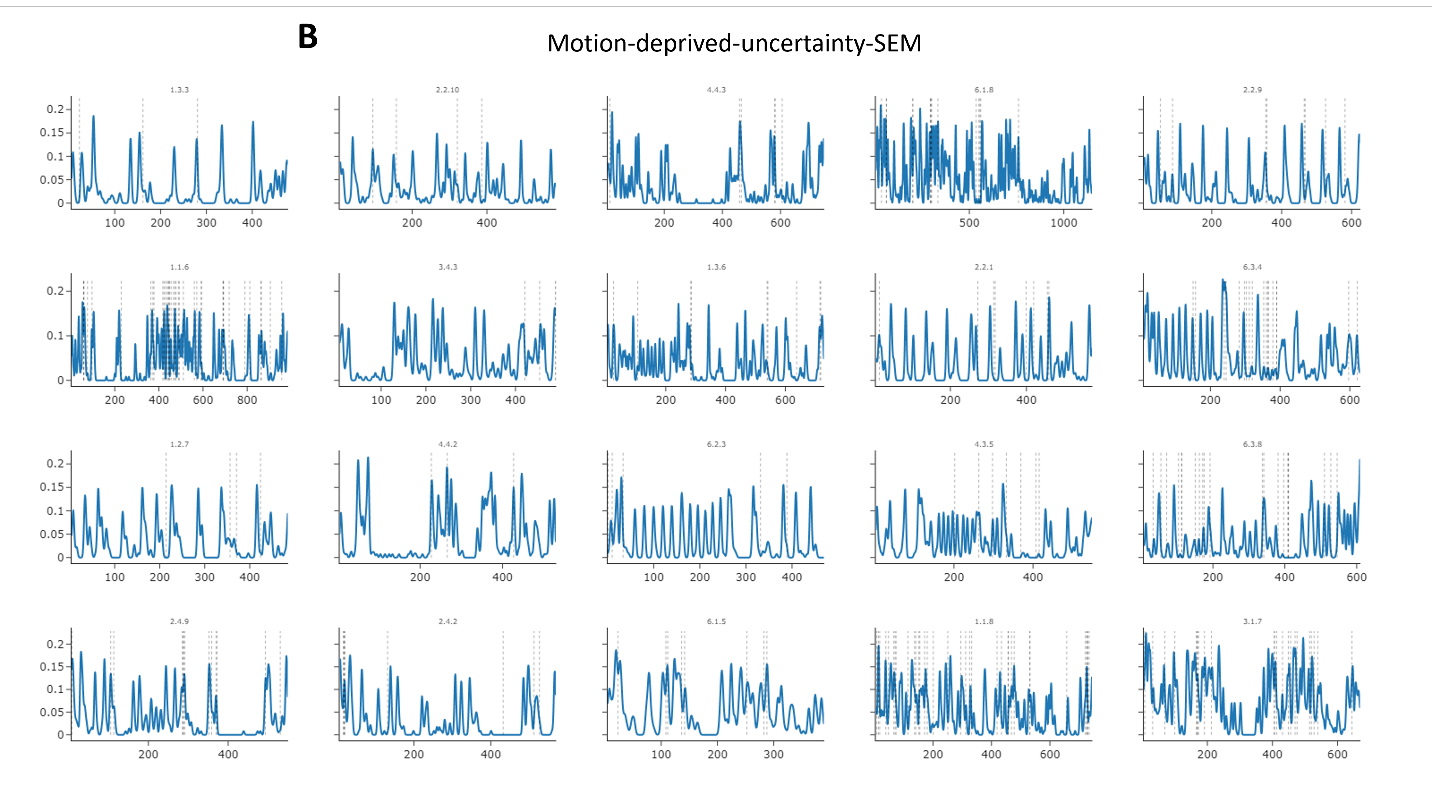


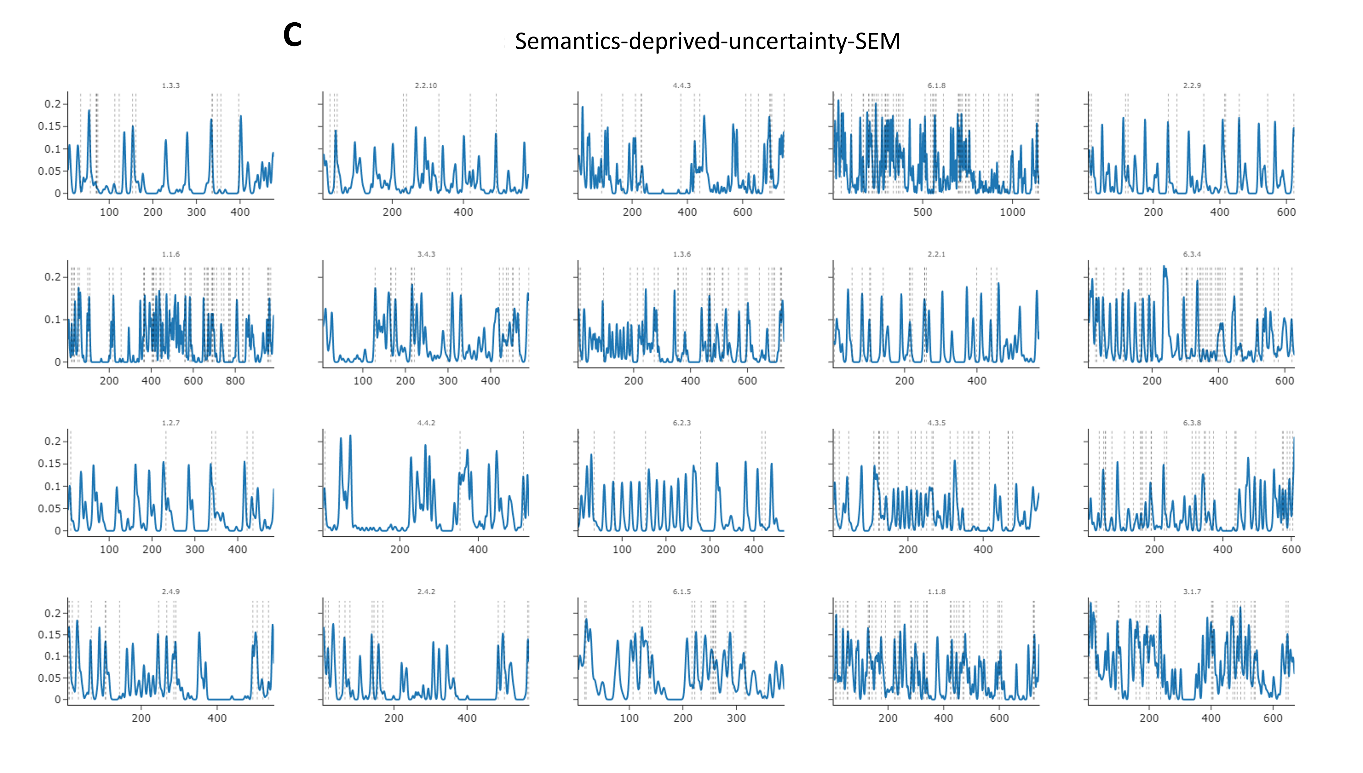


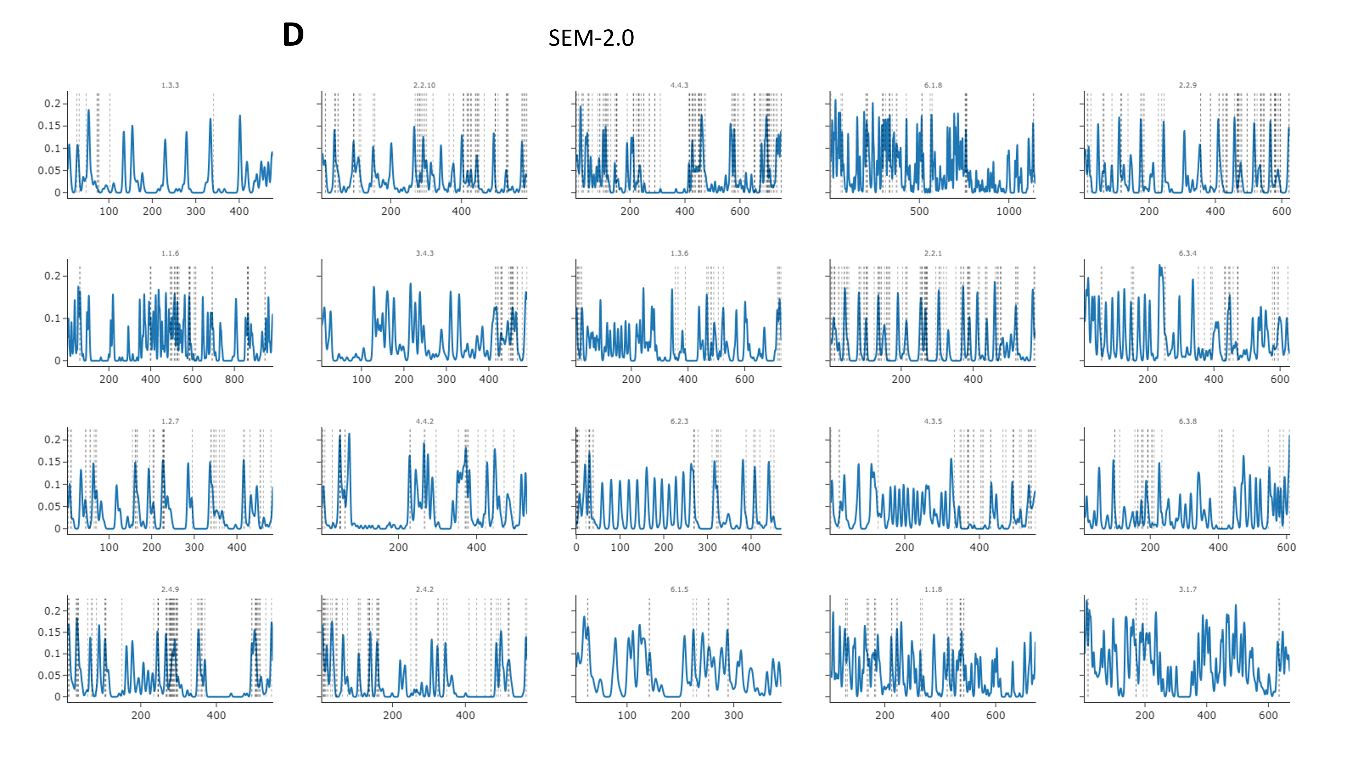


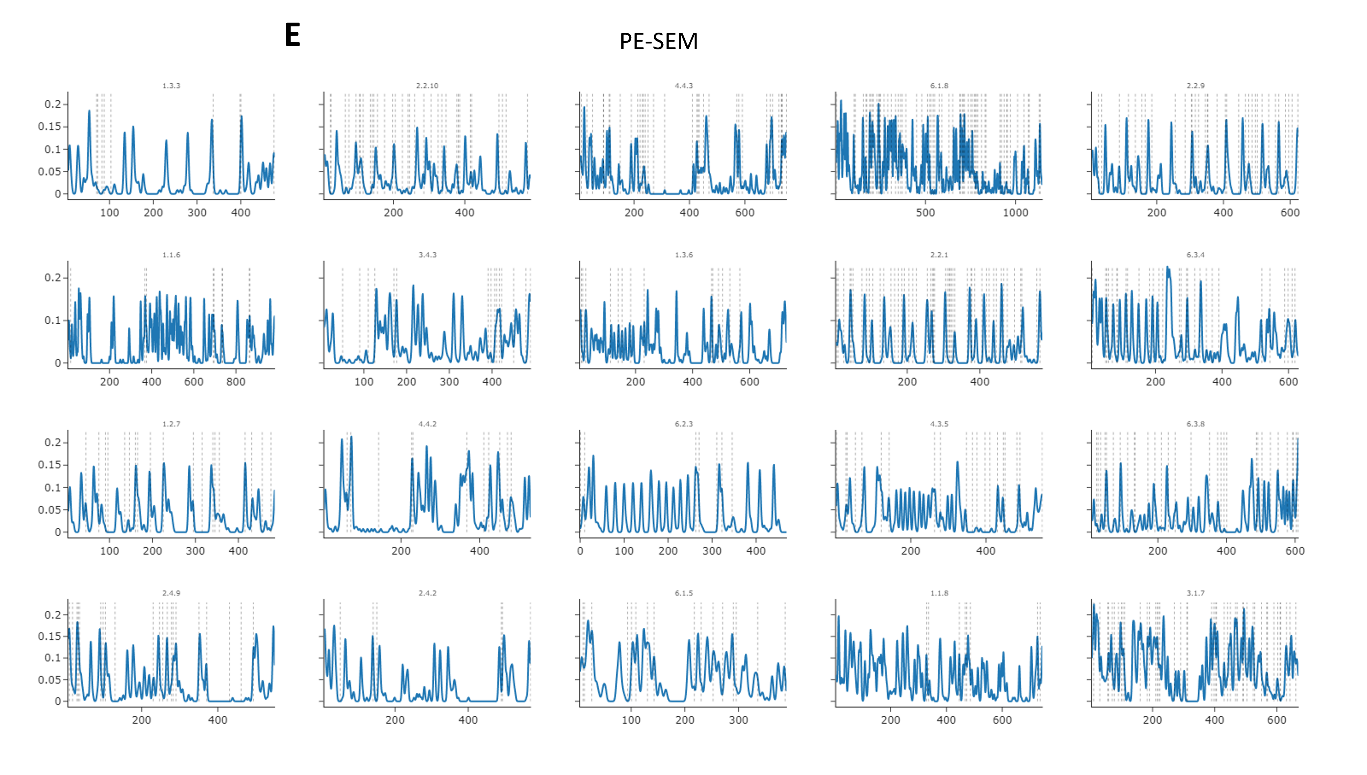


Fig. S12. Models’ event boundaries for one simulation. A-E: event boundaries from Uncertainty-SEM, motion-deprived-uncertainty-SEM, and semantic-deprived-uncertainty-SEM, SEM-2.0, and pe-SEM for all validation activities. Model-generated event boundaries are represented as dashed vertical lines, human fine-grain segmentation is represented as blue lines. The x-axis indicates the time (in seconds) within the activities and the y-axis indicates the human boundary probability. Each pane corresponds to each validation activities. Note that there were 8 simulations for each model, depicted here is only from one simulation.


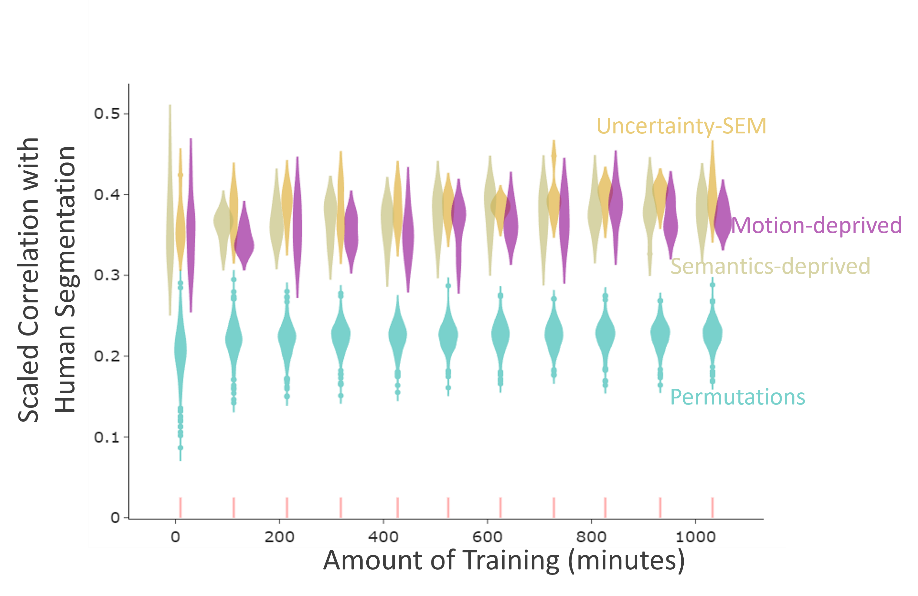


Fig. S13. Full Uncertainty-SEM’s segmentation agreement with humans is higher than deprived models’ segmentation agreement. Uncertainty-SEM denotes boundaries from uncertainty-SEM with full input features, semantics-deprived denotes boundaries from semantics-deprived model, and motion-deprived denotes boundaries from motion-deprived model. Permutations denotes permutated boundaries from all models. Each violin plot is a distribution of scaled point-biserial correlation for different initializations and training orders. The plotted distributions are jittered in the x dimension to reduce overlap; red ticks mark true sampled timepoints. Scaled correlations between deprived models and humans exceeds chance performance, and they are smaller than full uncertainty-SEM’s scaled correlations with humans.


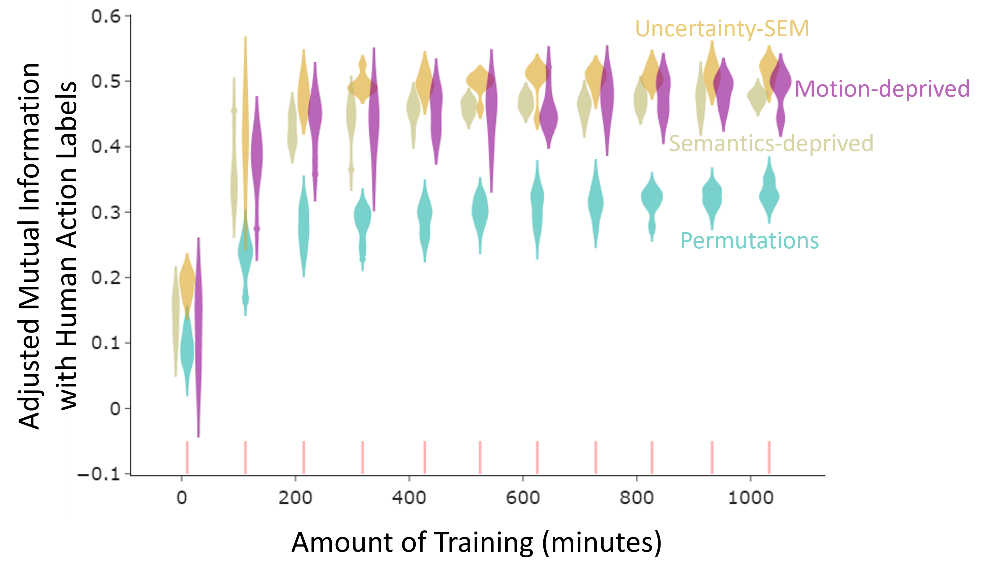


Fig. S14. Uncertainty-SEM categorization agreement with human action labels is higher than deprived models’ categorization agreement. Each violin plot is a distribution of adjusted mutual information scores for different initializations and training orders. Violins were dodged for visualization (vertical red ticks mark true sampled timepoints), even though all models have the same amount of training time (in minutes). Categorization agreement between deprived models and humans is greater than expected by chance.


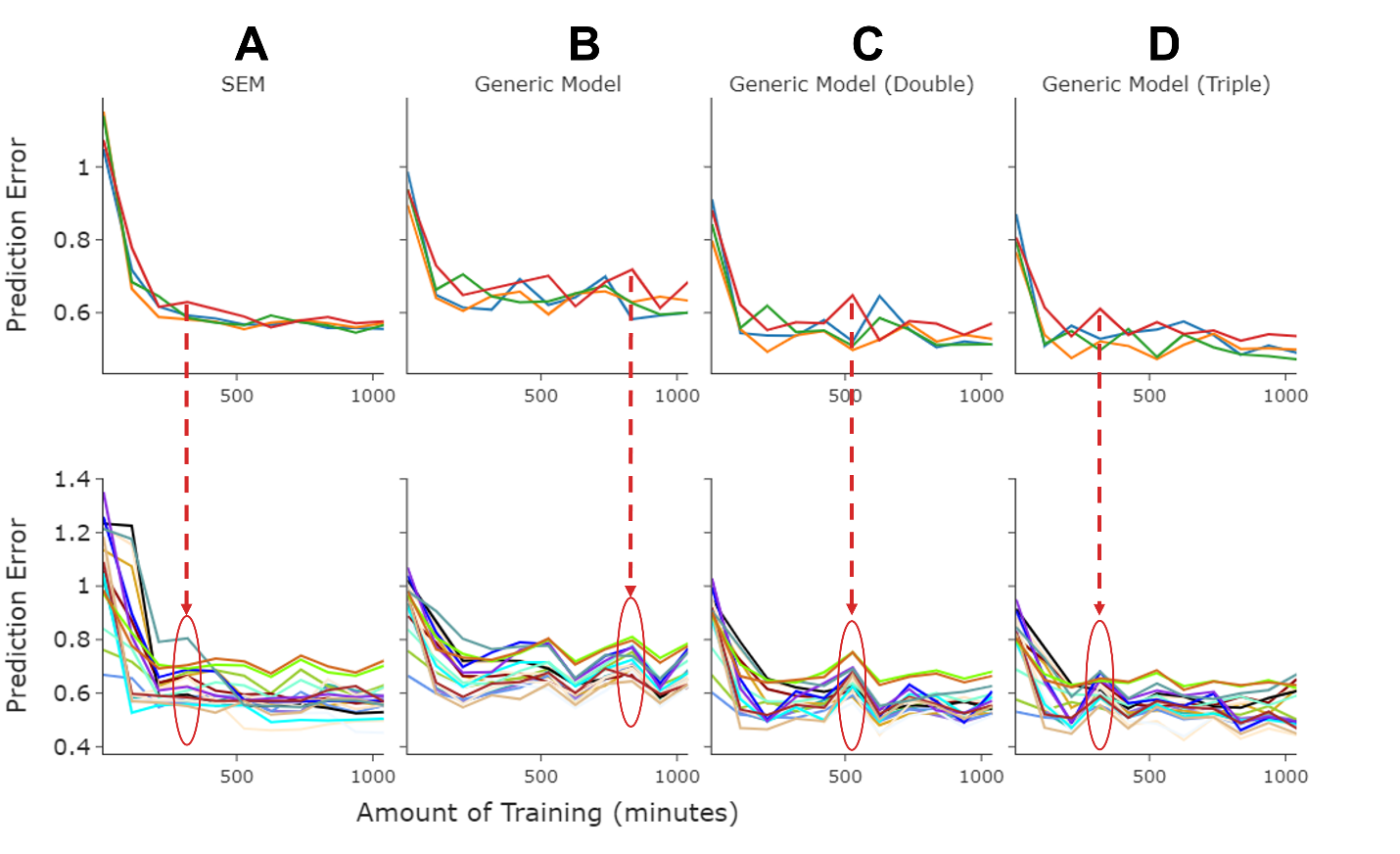


Fig. S15. Comparison of interference between SEM-2.0 and generic models. (A-D) Top: Mean prediction error for all validation activities across training for SEM-2.0, generic model with the same, double, and triple the number of hidden units. Four colors indicate four different simulations with different random weight initializations and different orders of training activities. Using discrete event schemas and a Bayesian updating process, SEM-2.0 reduces prediction error over the course of training. The generic model reduces prediction error over the course of training, to a level of prediction error slightly higher than SEM-2.0. Generic models with double and triple the number of hidden units can reduce errors lower than SEM-2.0; however, all generic models show greater interference from new learning (mean prediction error fluctuates across training). Bottom: Prediction errors for all validation activities across training for SEM-2.0, generic model with the same, double, and triple the number of hidden units, for the “red” simulation. SEM-2.0 shows interference around minute 300-th, with prediction errors for a couple of validation activities increasing. However, the generic model shows a higher degree of interference around minute 800-th, with almost all validation activities’ prediction errors increasing. The same pattern can be observed in the generic models with double and triple number of hidden units.


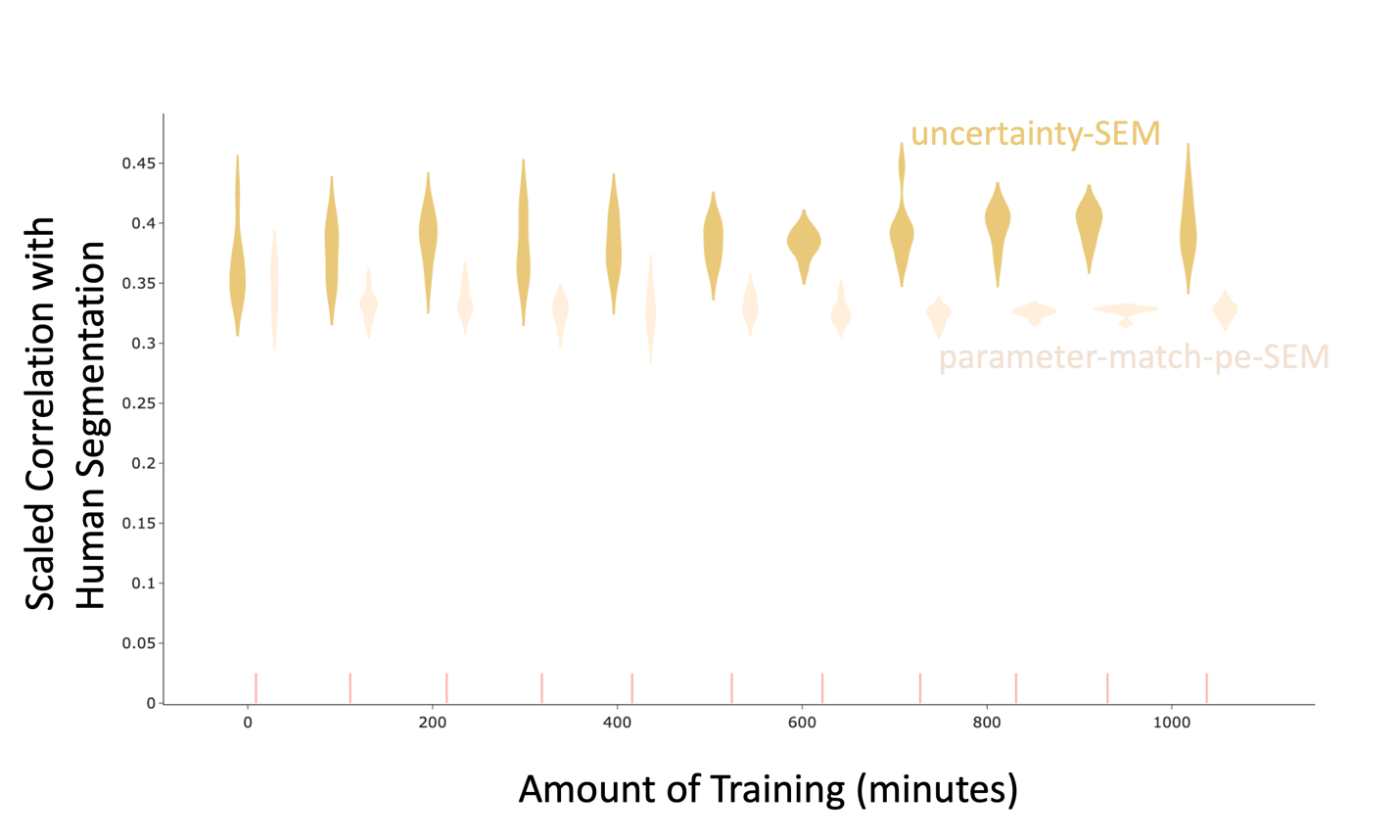


Fig. S16. PE-SEM whose parameters are the same as uncertainty-SEM’s parameters had lower segmentation agreement with human action labels than uncertainty-SEM did. Uncertainty-SEM identified similar numbers of events to fine-grain human segmentation.


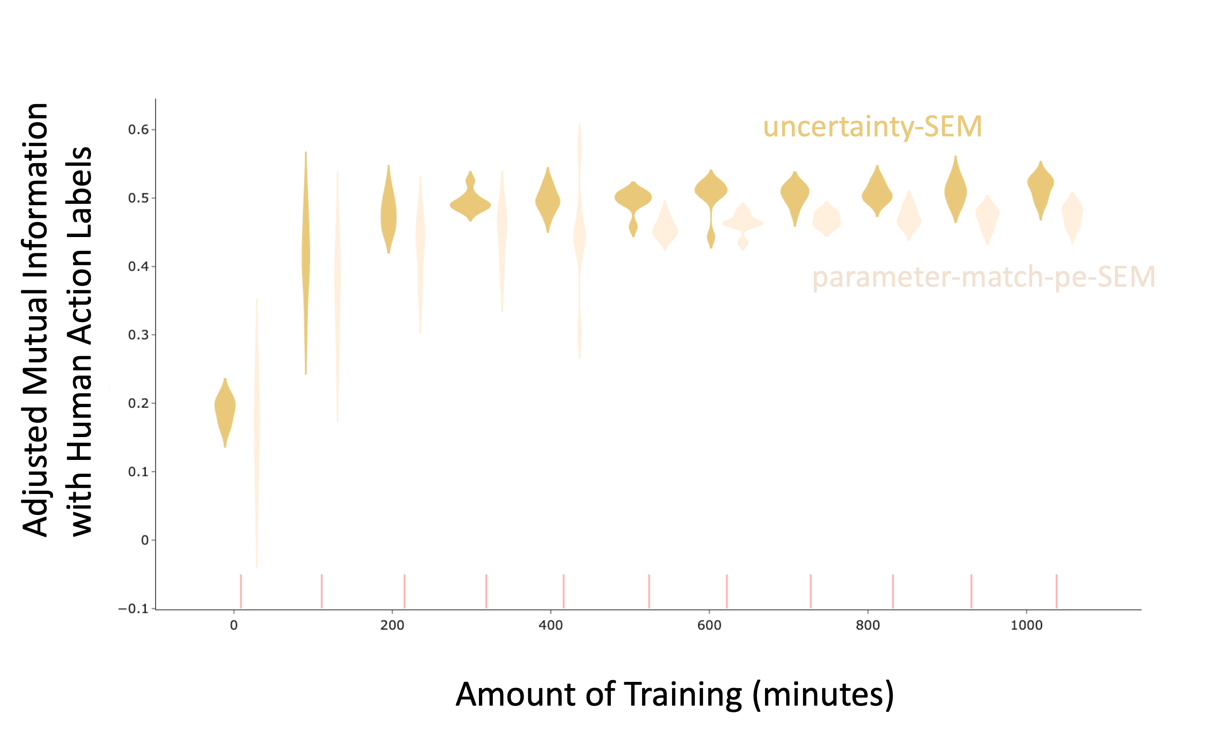


Fig. S17. PE-SEM whose parameters are the same as uncertainty-SEM’s parameters had lower categorization agreement with human action labels than uncertainty-SEM did. Uncertainty-SEM identified similar numbers of events to fine-grain human segmentation.


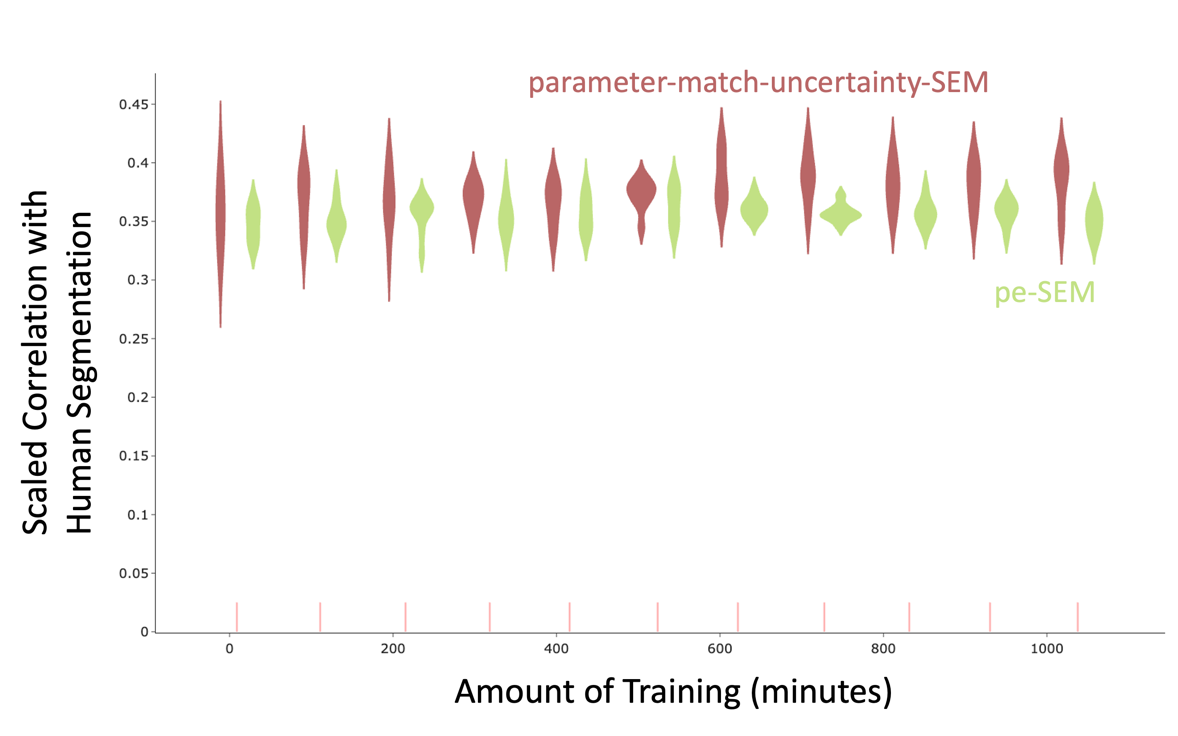


Fig. S18. Uncertainty-SEM whose parameters are the same as pe-SEM’s parameters had higher segmentation agreement with human event boundaries than pe-SEM did. PE-SEM identified similar numbers of events to fine-grain human segmentation.


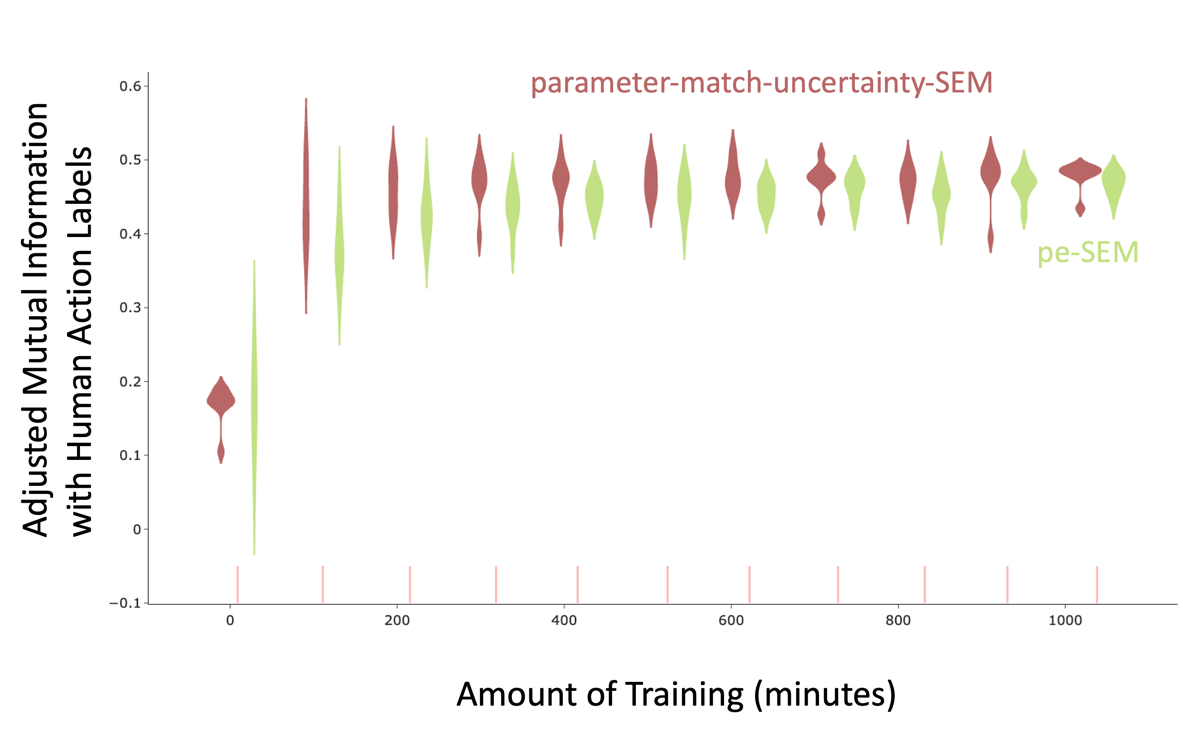


Fig. S19. Uncertainty-SEM whose parameters are the same as pe-SEM’s parameters had higher categorization agreement with human action labels than pe-SEM did. PE-SEM identified similar numbers of events to fine-grain human segmentation.


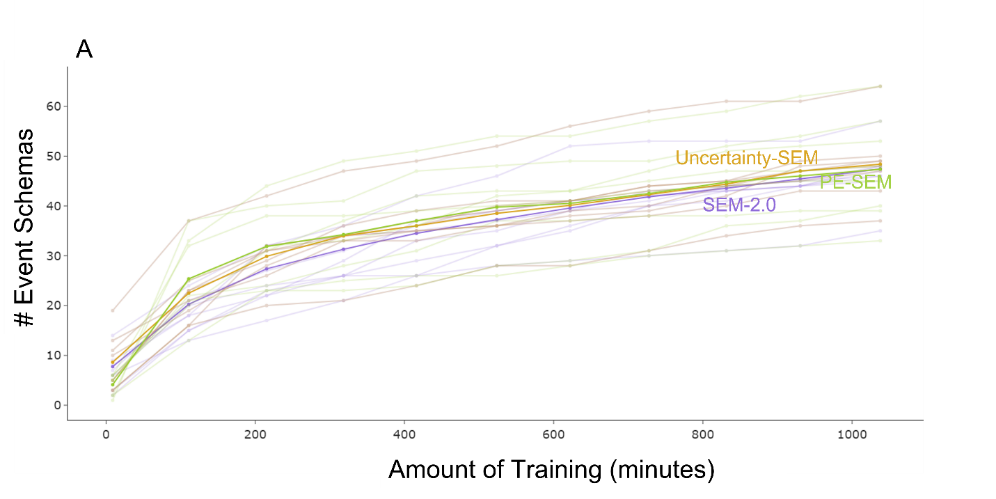

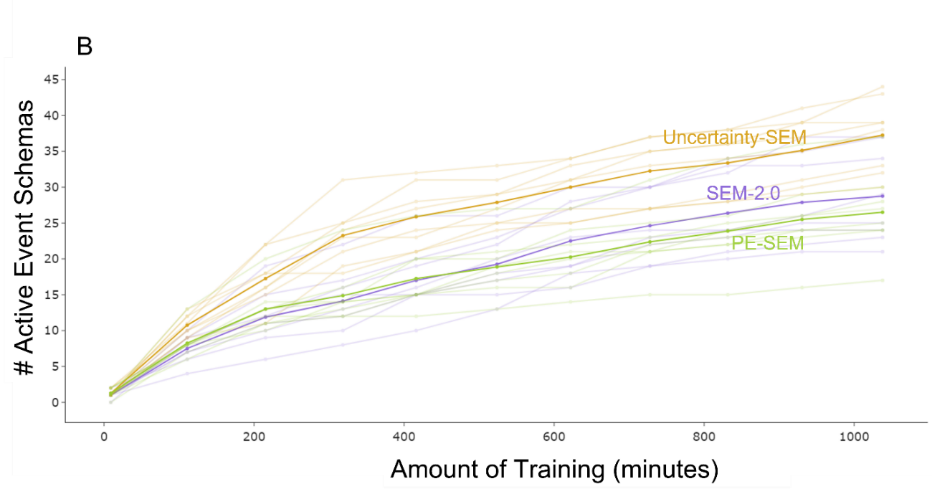


Fig. S20. Rate of schema generation and usage for three models. Same color lines are simulations of each model with different weight initializations and training orders. Shaded lines are for each simulation, and solid lines are the average of simulations for each model. A) All models created a similar number of schemas B) All models created many new event schemas early in training and reused these event schemas in the middle and late of training. Uncertainty-SEM has more active event schemas (being activated more than 300 seconds) than other models, despite spawning the same number of event schemas.


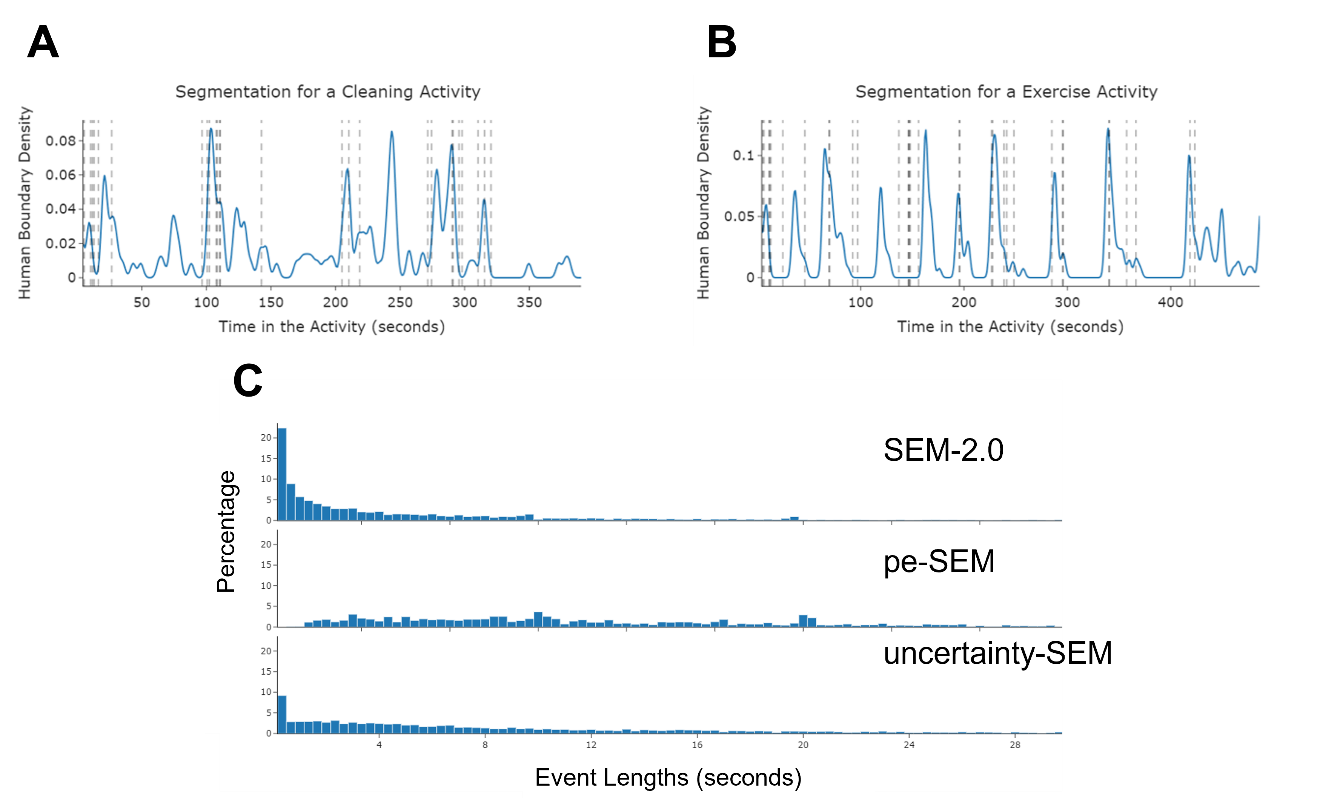


Fig. S21. The trained models show flurries of event model transitioning. Examples of SEM-2.0’s boundaries for (A) one “cleaning room” activity and (B) one “exercise” activity. These plots show the density of human-identified boundaries in blue, with vertical dotted lines for each time SEM-2.0 transitioned event models. For the cleaning activity, SEM-2.0 made flurries of switches around the 10th and 105th seconds. For the exercise activity, SEM-2.0 made flurries of switches around the 5th and 150th seconds. (C) Distribution of elapsed time between each model’s consecutive boundaries (event lengths). Durations longer than 30 seconds were not plotted. Approximately 36% of SEM-2.0’s and about 15% of uncertainty-SEM’s pairs of consecutive boundaries have durations have durations ≤1 second, indicating that SEM-2.0 and uncertainty-SEM made rapid switches within short intervals.

Table S1. Hyper-parameters that control SEM’s sensitivity to error, noise variance prior and degree of freedom.

| Noise variance prior (ν) | Degree of freedom for noise variance prior (τ) |
| --- | --- |
| 0.06 | 10 |

Table S2. Hyper-parameter values for the Adam optimization algorithm used to train the RNN and RNN’s parameters. All simulations used the same optimization hyper-parameters.

| Β_1_ | β_2_ | ε | Decay | n_epochs | n_hidden_units | n_hidden_layer |
| --- | --- | --- | --- | --- | --- | --- |
| 0.9 | 0.999 | 1e-08 | 0 | 10 | 16 | 2 |

| Model | Learning Rate | Stickiness | Concentration | Threshold |
| --- | --- | --- | --- | --- |
| SEM-2.0 | 1e-3 | 1e5 | 1e-1 | N/A |
| Uncertainty-SEM | 1e-3 | 5e-3 | 1e0 | 2.5e-3 |
| PE-SEM | 1e-3 | 5e0 | 2e-2 | 4.3e-1 |

Table S3. Models’ hyper-parameters.

SI References

1. M. A. Bezdek, T. T. Nguyen, C. S. Hall, T. S. Braver, A. F. Bobick, J. M. Zacks, The multi-angle extended three-dimensional activities (META) stimulus set: A tool for studying event cognition. *Behav Res*, doi: 10.3758/s13428-022-01980-8 (2022).

2. N. T. Franklin, K. A. Norman, C. Ranganath, J. M. Zacks, S. J. Gershman, Structured event memory: A neuro-symbolic model of event cognition. *Psychological Review* **127**, 327–361 (2020).
